# Supplementary material for: RBP4-STRA6 Pathway Drives Cancer Stem Cell Maintenance and Mediates High-Fat Diet-Induced Colon Carcinogenesis
Source: Stem Cell Reports. 2017 Jul 6;9(2):438–50. doi: 10.1016/j.stemcr.2017.06.002 (PMC5549806; doi:10.1016/j.stemcr.2017.06.002)
Supplement: Document S1. Figures S1–S5 [file mmc1.pdf]

Stem Cell Reports, Volume *9*

Supplemental Information

RBP4-STRA6 Pathway Drives Cancer Stem Cell Maintenance and Mediates High-Fat Diet-Induced Colon Carcinogenesis

Sheelarani Karunanithi, Liraz Levi, Jennifer DeVecchio, George Karagkounis, Ofer Reizes, Justin D. Lathia, Matthew F. Kalady, and Noa Noy

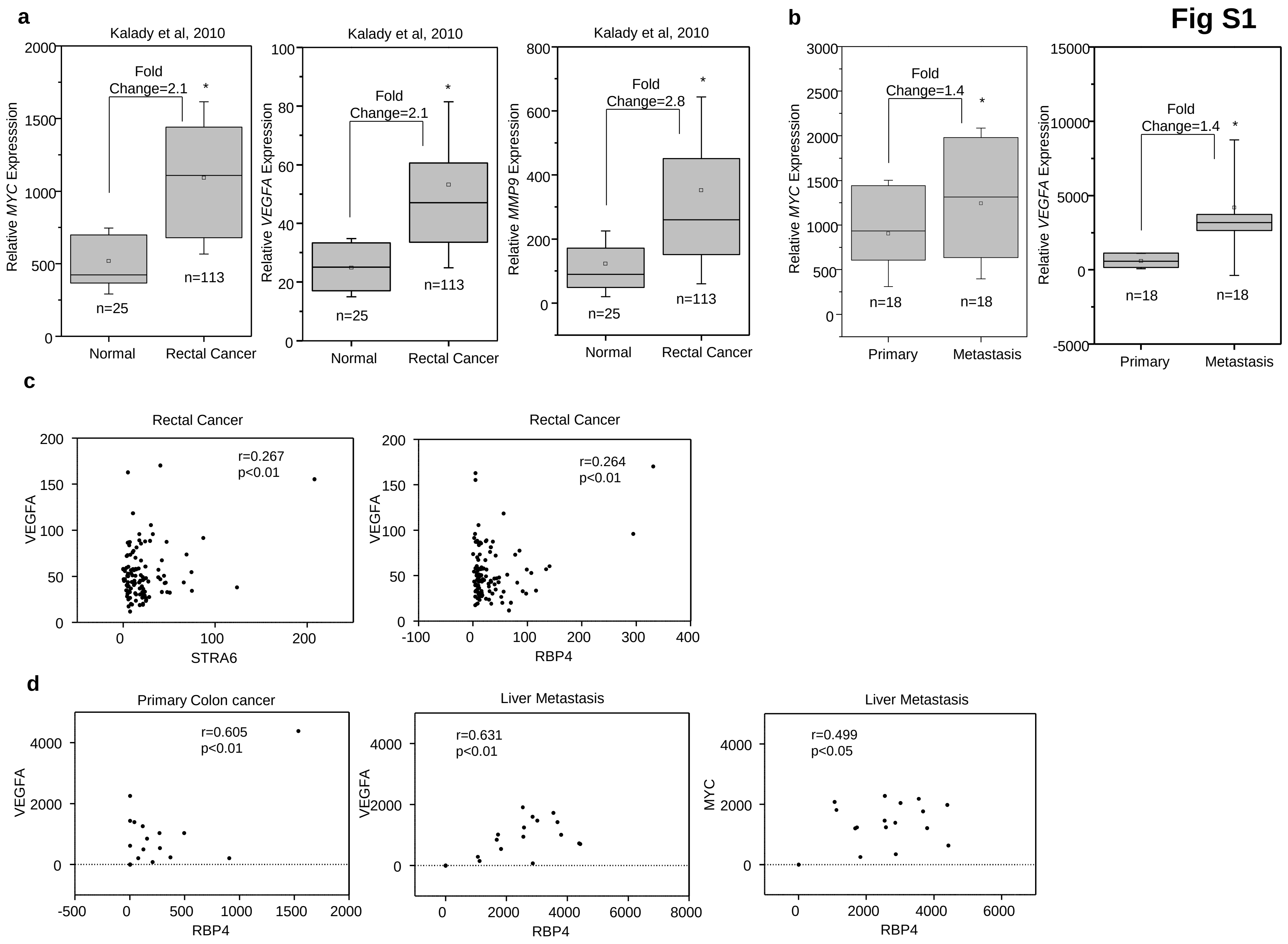

Fig S1
a
Kalady et al, 2010
Fold Change=2.1
*
Relative MYC Expresssion
n=25
n=113
Normal
Rectal Cancer
Kalady et al, 2010
Fold Change=2.1
Relative VEGFA Expression
n=25
n=113
Normal
Rectal Cancer
Kalady et al, 2010
Fold Change=2.8
Relative MMP9 Expression
n=25
n=113
Normal
Rectal Cancer
b
Fold Change=1.4
Relative MYC Expresssion
n=18
n=18
Primary
Metastasis
Fold Change=1.4
Relative VEGFA Expression
n=18
n=18
Primary
Metastasis
c
Rectal Cancer
r=0.267
p<0.01
VEGFA
STRA6
Rectal Cancer
r=0.264
p<0.01
VEGFA
RBP4
d
Primary Colon cancer
r=0.605
p<0.01
VEGFA
RBP4
Liver Metastasis
r=0.631
p<0.01
VEGFA
RBP4
Liver Metastasis
r=0.499
p<0.05
MYC
RBP4

Fig S2

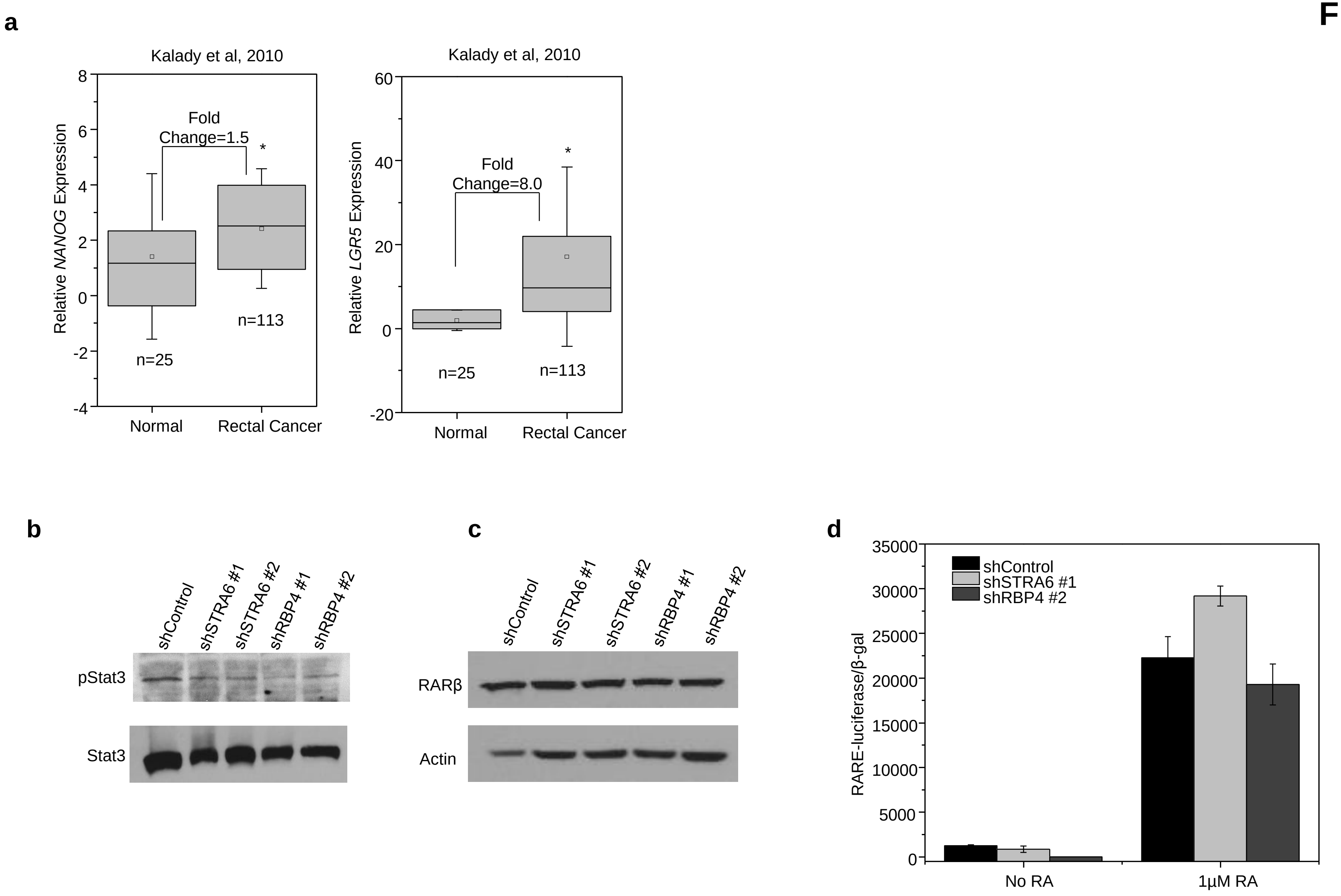
a
Kalady et al, 2010
Relative NANOG Expression
Fold Change=1.5
*
n=25
n=113
Normal
Rectal Cancer
8
6
4
2
0
-2
-4
Kalady et al, 2010
Relative LGR5 Expression
Fold Change=8.0
*
n=25
n=113
Normal
Rectal Cancer
60
40
20
0
-20
b
shControl
shSTRA6 #1
shSTRA6 #2
shRBP4 #1
shRBP4 #2
pStat3
Stat3
c
shControl
shSTRA6 #1
shSTRA6 #2
shRBP4 #1
shRBP4 #2
RARβ
Actin
d
shControl
shSTRA6 #1
shRBP4 #2
RARE-luciferase/β-gal
35000
30000
25000
20000
15000
10000
5000
0
No RA
1μM RA

Fig S3

a

SW480	Number of spheres positive wells/ Number of wells			
	Number of cells per well			
	1	2	5	10
shControl	8/24	13/24	21/24	24/24
shSTRA6 #1	3/24	4/24	12/24	16/24
shSTRA6 #2	3/24	5/24	14/24	19/24
shRBP4 #1	2/24	4/24	6/24	13/24
shRBP4 #2	4/24	7/24	9/24	12/24

b

SW480	Number of tumors / Number of injected mice				
	Number of cells per injection				
	$2.5*10^5$	$1.5*10^4$	$1*10^4$	5000	1500
shControl			4/5	2/5	1/5
shSTRA6 #1			2/5	0/5	0/5
shRBP4 #1	5/5	2/5			0/6

c

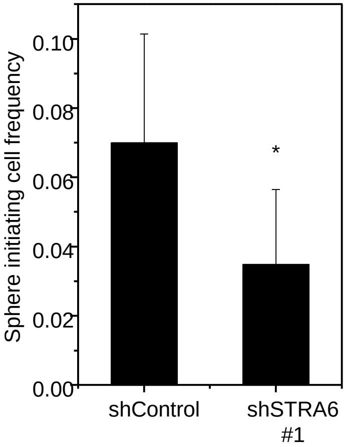

SW480	Number of spheres positive wells/ Number of wells					Sphere initiating-cell frequency		Fold Change	p value
	Number of cells per well					Estimate	95% Confidence Interval		
	1	2	5	10	20				
shControl	3/24	4/24	10/24	4/12	8/12	1/14.3	(1/9.86-1/20.7)		
shSTRA6 #1	1/24	2/24	2/24	5/12	6/12	1/28.7	(1/17.7-1/46.7)	2	p<0.05

d

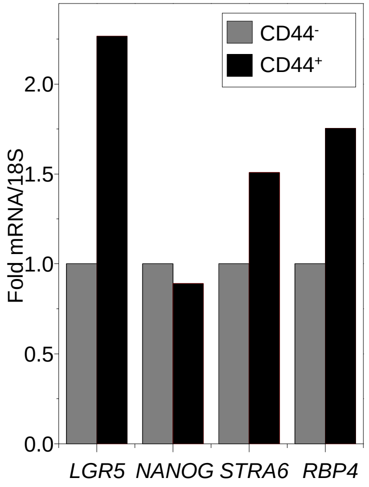

e

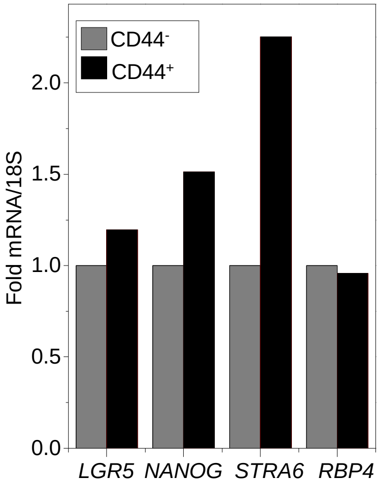

Fig S4

a

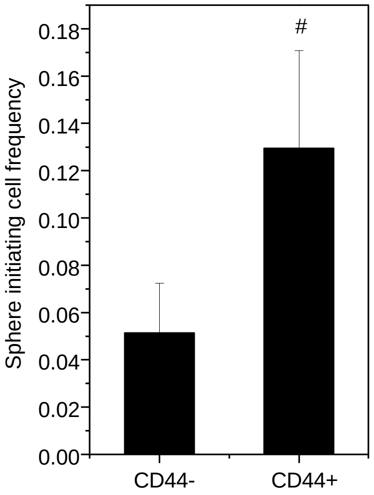

b

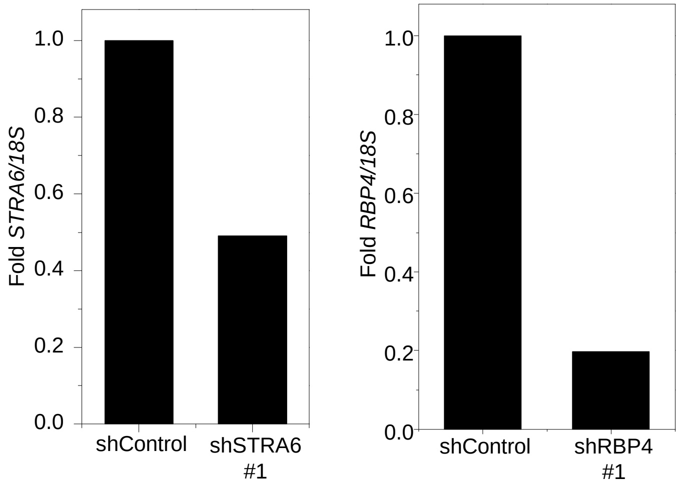

c

PDX656	Number of spheres positive wells/ Number of wells
	Number of cells per well<br>1 5 10 20
shControl	7/24 13/24 22/24 24/24
shSTRA6 #1	3/24 10/24 14/24 20/24
shControl	7/24 14/24 19/24 21/24
shRBP4 #1	3/24 7/24 12/24 18/24

Fig S5

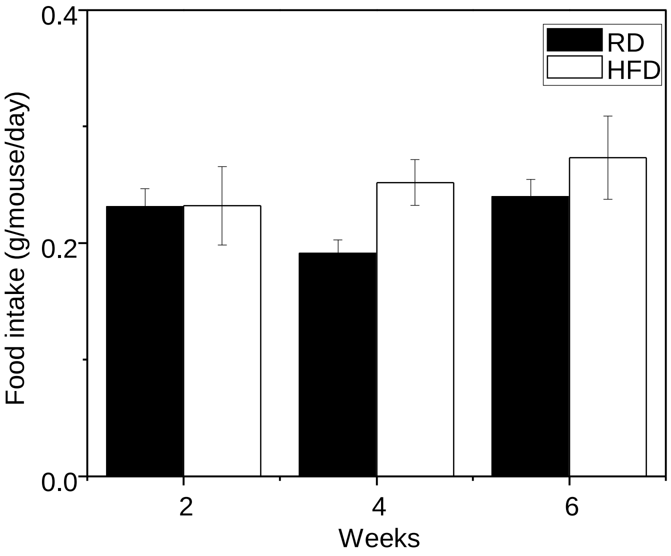

Supplemental Figure legends:

Figure S1: Related to Figure 1. JAK-STAT target gene expression correlate to RBP4-STRA6 pathway. a) Levels of STAT target genes, MYC, VEGFA and MMP9 in rectal cancer patients compared to normal tissue. * $p<0.01$ b) Analysis of expression levels of MYC and VEGFA in matched primary and metastatic colon cancer samples. *$p<0.05$ c) Correlation between expression levels of VEGFA and STRA6 or RBP4 in rectal cancer patients. Inset shows Pearson coefficient 'r' and p value. d) Correlation between expression levels of VEGFA and RBP4 in primary colon cancer and between VEGFA or MYC and RBP4 in matched liver metastasis patients are presented with Pearson coefficient 'r'.

Figure S2: Related to Figures 1 &2. RBP4-STRA6 pathway is related to colon cancer stemness. a) NANOG and LGR5 expression is upregulated in rectal cancer patients. *$p<0.01$. b) Immunoblots showing phospho-STAT3 and total STAT3 levels in SW480 cells stably knockdown for STRA6 or RBP4. c) Immunoblot showing unchanged protein levels of retinoic acid (RA) target gene, RARβ in SW480 cells stably expressing control, STRA6 or RBP4 shRNAs. Actin is used as a loading control. d) Activation of RARα in response to treatment with 1µM retinoic acid in SW480 stable lines was measured using transactivation assays. Cells were transfected with RAR-response element luciferase, RARα and β-gal constructs before treatments. Luciferase activity in response to treatment was normalized to β-galactosidase levels. Data are mean±SE of 3 biological replicates . $p<0.05$ (comparisons are between untreated and treated samples).

Figure S3: Related to Figure 4. Knockdown of STRA6 or RBP4 decrease CSC frequency. a) Limiting dilution assay in indicated SW480 lines shown in Fig 4B. Dose of cells seeded and the resulting frequency of sphere formation are tabulated. b) Tumor initiating cell frequency at indicated doses of SW480 cell injections from *in vivo* limiting dilution assay in Fig 4C. Frequency of tumor formation at respective dose of cells are shown in the table. c) *In vitro* limiting dilution assay of cells dissociated from SW480 tumors in Fig 4C expressing control or STRA6 shRNAs. Frequency estimates were calculated from 24 biological replicates of varying cell doses. Sphere re-initiation frequency calculated using ELDA software is plotted with error bars representing 95% confidence interval. *$p<0.05$. Stem cell frequency estimates within confidence intervals are shown in the table. d) Tumors from SW480 shControl injections were dissociated, stained with CD44-FITC antibody and sorted on BD FACS ARIA II. RNA levels of *STRA6*, *RBP4*, *NANOG* and *LGR5* in CD44 negative and positive populations are shown. Each bar represents expression analysis of cells dissociated after pooling several tumors from mice (n=5). e) Expression levels of *STRA6*, *RBP4*, *NANOG* and *LGR5* in CD44 negative and positive populations of PDX656 maintained as non-adherent spheres in culture.

Figure S4: Related to Figures 5. Knockdown efficiencies in a PDX. a) Sphere-initiating cell frequency of CD44 negative and positive cells sorted from cultured PDX 656 cells. Frequency estimates were calculated from 24 biological replicates of varying cell doses. Graph represents the fraction of sphere-forming stem cells and error bars represent 95% confidence intervals. #$p<0.01$. b) Knockdown efficiency of STRA6 (left) and RBP4 (right) shRNA in PDX 656 shown by differences in mRNA levels. c) Table shows dose of cells and frequency of sphere formation in a limiting dilution assay in PDX 656 performed in Fig 5A.

Figure S5: Related to Figures 6. Food intake is unaffected by diet. Food intake in mice fed a regular or high-fat diet in Fig 6A. Amount of food consumed normalized to body weights is shown during 2, 4 and 6 weeks after the mice were placed on the dietary regimen. n= 5 mice.
