# Supplementary material for: RBP4-STRA6 Pathway Drives Cancer Stem Cell Maintenance and Mediates High-Fat Diet-Induced Colon Carcinogenesis
Source: Stem Cell Reports. 2017 Jul 6;9(2):438–50. doi: 10.1016/j.stemcr.2017.06.002 (PMC5549806; doi:10.1016/j.stemcr.2017.06.002)
Supplement: Document S2. Article plus Supplemental Information [file mmc2.pdf]

Stem Cell Reports
Article
OPEN ACCESS

RBP4-STRA6 Pathway Drives Cancer Stem Cell Maintenance and Mediates High-Fat Diet-Induced Colon Carcinogenesis

Sheelarani Karunanithi,[1,*] Liraz Levi,[6] Jennifer DeVecchio,[3] George Karagkounis,[3,4] Ofer Reizes,[1,5,6] Justin D. Lathia,[1,5,6] Matthew F. Kalady,[3,4,5,*] and Noa Noy[1,2,5,6]
[1]Department of Cellular & Molecular Medicine, Lerner Research Institute, Cleveland Clinic, Cleveland, OH 44195, USA
[2]Department of Nutrition and Case Comprehensive Cancer Center, Case Western Reserve University, Cleveland, OH 44106, USA
[3]Department of Stem Cell Biology and Regenerative Medicine, Lerner Research Institute, Cleveland Clinic, Cleveland, OH 44195, USA
[4]Department of Colorectal Surgery, Digestive Disease Institute, Cleveland Clinic, Cleveland 44195, OH, USA
[5]Department of Molecular Medicine, Cleveland Clinic Lerner College of Medicine at Case Western Reserve University, Cleveland, OH 44195, USA
[6]Case Comprehensive Cancer Center, Case Western Reserve University, Cleveland, OH 44106, USA
*Correspondence: srk115@case.edu (S.K.), kaladym@ccf.org (M.F.K.)
http://dx.doi.org/10.1016/j.stemcr.2017.06.002

SUMMARY

The transmembrane protein, STRA6, functions as a vitamin A transporter and a cytokine receptor when activated by vitamin A-bound serum retinol binding protein 4 (RBP4). STRA6 activation transduces a JAK2-STAT3 signaling cascade and promotes tumorigenesis in a xenograft mouse model of colon cancer. We show here that RBP4 and STRA6 expression is associated with poor oncologic prognosis. Downregulating STRA6 or RBP4 in colon cancer cells decreased the fraction of cancer stem cells and their sphere and tumor initiation frequency. Furthermore, we show that high-fat diet (HFD) increases LGR5 expression and promotes tumor growth in a xenograft model independent of obesity. HFD increased STRA6 levels, and downregulation of STRA6 delays and impairs tumor initiation, tumor growth, and expression of stemness markers. Together, these data demonstrate a key role of STRA6 and RBP4 in the maintenance of colon cancer self-renewal and that this pathway is an important link through which consumption of HFD contributes to colon carcinogenesis.

INTRODUCTION

Colorectal cancer is the third most common cancer in the United States, with more than 130,000 patients reported yearly representing nearly 8% of newly identified cancer cases (Howlader et al., 2016). Colon cancer arises as a result of a combination of several genetic, epigenetic, and environmental causes. The most common genetic alterations include mutations in *KRAS*, *BRAF*, *TP53*, and members of Wnt and transforming growth factor β pathways (Lao and Grady, 2011; Walther et al., 2009). Epigenetic changes such as DNA methylation, histone modifications, and microsatellite and chromosomal instability, among others, have been linked to colorectal cancer (Colussi et al., 2013; van Engeland et al., 2011). Among the environmental risk factors associated with colon cancer, influence of diet has been widely studied and is associated with nearly 80% of colon cancer cases (Nystrom and Mutanen, 2009). Obesity and high-fat and western diets are thought to increase colon cancer risk and recurrence (Beyaz et al., 2016; Newmark et al., 2001; Ning et al., 2010; Reddy, 2002). However, detailed studies examining the direct impact of individual dietary components (not obesity per se) and the related pathways are lacking.

We have recently shown a role for vitamin A signaling in promoting colorectal tumor growth (Berry et al., 2014). Vitamin A, or retinol, circulates in blood bound to a serum retinol binding protein, RBP4 (Noy, 2000). Retinol is a lipophilic molecule that can readily diffuse into cells through the plasma membrane toward a concentration gradient. Retinol can also be transported through a cell surface protein, Stimulated by Retinoic Acid 6 (STRA6) (Berry et al., 2013; Kawaguchi et al., 2007). Yet a lack of STRA6 does not affect vitamin A homeostasis in most tissues except those where it is very highly expressed, such as the eye (Berry et al., 2013). STRA6 is a dimeric transmembrane protein that functions as a vitamin A transporter as well as a cytokine signaling receptor (Berry et al., 2011; Chen et al., 2016; Kawaguchi et al., 2007). Binding of retinol bound RBP4 (holo-RBP4) to STRA6 leads to activation of a JAK2-STAT3 signaling cascade. JAK2 phosphorylates STRA6 at Y643 in its cytosolic tail and STAT3 is recruited to sites of activated and phosphorylated STRA6. JAK2 phosphorylation of STAT3 causes dimerization and nuclear translocation of STAT3 where it functions as a transcription factor (Berry et al., 2011, 2012).

STAT3 is a known driver of oncogenesis (Bromberg et al., 1999) and indeed, activation of STRA6 signaling was demonstrated to promote tumor progression in a STAT3-dependent manner using a xenograft model of colon cancer (Berry et al., 2014). RBP4 and STRA6 are both upregulated in colon cancer compared with normal colon (Berry et al., 2014; Szeto et al., 2001). Interestingly, the RBP4-STRA6 pathway is associated with high-fat diet (HFD)-induced metabolic phenotype, and inactivation of this pathway improves insulin resistance (Berry et al., 2013;

 © 2017 the Cleveland Clinic.
This is an open access article under the CC BY license (http://creativecommons.org/licenses/by/4.0/).

Yang et al., 2005). Obesity is known to increase serum RBP4 levels (Yang et al., 2005), and in a recent study high serum RBP4 levels were correlated to increased colon adenoma risk (Abola et al., 2015). However, a relationship between RBP4-STRA6 pathway and HFD-mediated risk of colon cancer is not established.

Tumor initiation and tumor heterogeneity are driven in part by a population of self-renewing cells constituting ~1% of the bulk of the tumor called the cancer stem cells (CSCs) (Kreso and Dick, 2014; Vaiopoulos et al., 2012; Wicha et al., 2006). Differences in genetic, epigenetic, and environmental responses between tumor cells underlie tumor heterogeneity (Dick, 2008), as seen in several solid tumors including colon cancer (Wicha et al., 2006; Zeuner et al., 2014). CSCs have also been demonstrated to promote tumor recurrence, metastasis, and therapeutic resistance, and have the capacity to repopulate a niche as a heterogeneous tumor from a small number of cells (Lotti et al., 2013; Merlos-Suarez et al., 2011; Vaiopoulos et al., 2012). The complete network of pathways that regulates CSC self-renewal is not known.

Although the RBP4-STRA6 pathway has been studied in colon cancer tumorigenesis, the exact mechanism by which this pathway induces neoplastic changes is not clear. The effect of RBP4 on tumor growth has not been studied, and whether this pathway affects tumor initiation is unknown. We show here that STRA6 and RBP4 are necessary for optimal expression of stemness markers. The RBP4-STRA6 pathway is required for maintenance of the colon CSC pool and tumor initiation. Furthermore, our results establish the RBP4-STRA6 pathway as a link between HFD feeding and colon carcinogenesis.

RESULTS

STRA6 and RBP4 Expression Are Associated with Poor Prognosis of Colorectal Cancer

Analysis of colorectal cancer-free survival data present in the Cancer Genome Atlas (TCGA) available through cBioPortal (Cerami et al., 2012; Gao et al., 2013) showed a survival disadvantage when STRA6 or RBP4 was highly expressed (hazard ratio = 1.929, $p < 0.05$) (Figure 1A). Similarly to previously published data (Berry et al., 2014; Szeto et al., 2001), STRA6 mRNA expression levels were found to be markedly higher in samples collected from colon cancer (Figure 1B) and also in rectal cancer (Figure 1C) patients (for methods see Kalady et al., 2010) compared with the normal tissues. Neoadjuvant chemoradiotherapy (nCRT) is used to treat locally advanced rectal cancer patients. Interestingly, microarray analysis of a cohort of patients (Gantt et al., 2014) who responded incompletely or partially to nCRT showed higher levels of STRA6 expression compared with complete responders (Figure 1D).

Microarray analysis was extended to patient samples with specific clinical phenotypes. Matched primary colorectal cancer specimens and corresponding liver metastases were evaluated. Also, primary rectal cancers with or without 3-year recurrence of disease were studied (Kalady et al., 2010). RBP4 expression was elevated in colon cancer metastases compared with primary tumor (Figure 1E) and in patients who developed recurrent rectal cancer (Figure 1F). We further investigated whether RBP4 expression was associated with aggressive presentations of colorectal cancer using classifications based on low or stable microsatellite instability and constitutively active *KRAS* mutations. Microarray analysis of these two datasets (Hogan et al., 2015a; Sanchez et al., 2009) showed that RBP4 expression was significantly upregulated in patient datasets that carry low or stable microsatellite instability (Figure 1G) or *KRAS* mutations (Figure 1H). To delineate the contributions of serum versus autocrine secretion of RBP4 in the tumor microenvironment, we measured serum levels of RBP4 in a subset of patients from the KRAS wild-type and mutant groups. There was no difference in the serum RBP4 levels between the two groups (Figure 1I).

We have previously shown that the RBP4-STRA6 pathway can activate JAK-STAT phosphorylation (Berry et al., 2011) and its target genes MYC, matrix metalloproteinase 9 (MMP9), and vascular endothelial growth factor A (VEGFA) respond to this activation (Berry et al., 2014). Therefore, we analyzed these datasets for differential expression of JAK-STAT target genes. We found that MMP9, MYC, and VEGFA were upregulated (Figure S1A) in the rectal cancer group compared with normal tissue (Kalady et al., 2010). In the same dataset, there was also a significant but weak, positive correlation of VEGFA with STRA6 ($r = 0.267$) and RBP4 expression ($r = 0.264$) (Figure S1C). MYC and VEGFA levels were also increased in metastatic colon cancer cohort compared with primary tumor (Figure S1B), similar to RBP4 (Figure 1E). A moderate positive correlation of RBP4 was observed with VEGFA in the primary colon cancer ($r = 0.605$) and with VEGFA ($r = 0.631$) and MYC ($r = 0.499$) in liver metastases (Figure S1D). Together, these results indicate a strong correlation between the RBP4-STRA6 pathway and colorectal cancer. Moreover, the association of STRA6 and RBP4 expression with metastasis, tumor recurrence, and therapeutic resistance suggests a role for these proteins in regulating cancer-initiating cells.

STRA6 and RBP4 Regulate Pro-survival Properties

To examine the effect of STRA6 and RBP4 on colon cancer growth we generated, using lentiviral short hairpin RNA (shRNA), SW480 colon adenocarcinoma cell lines in which

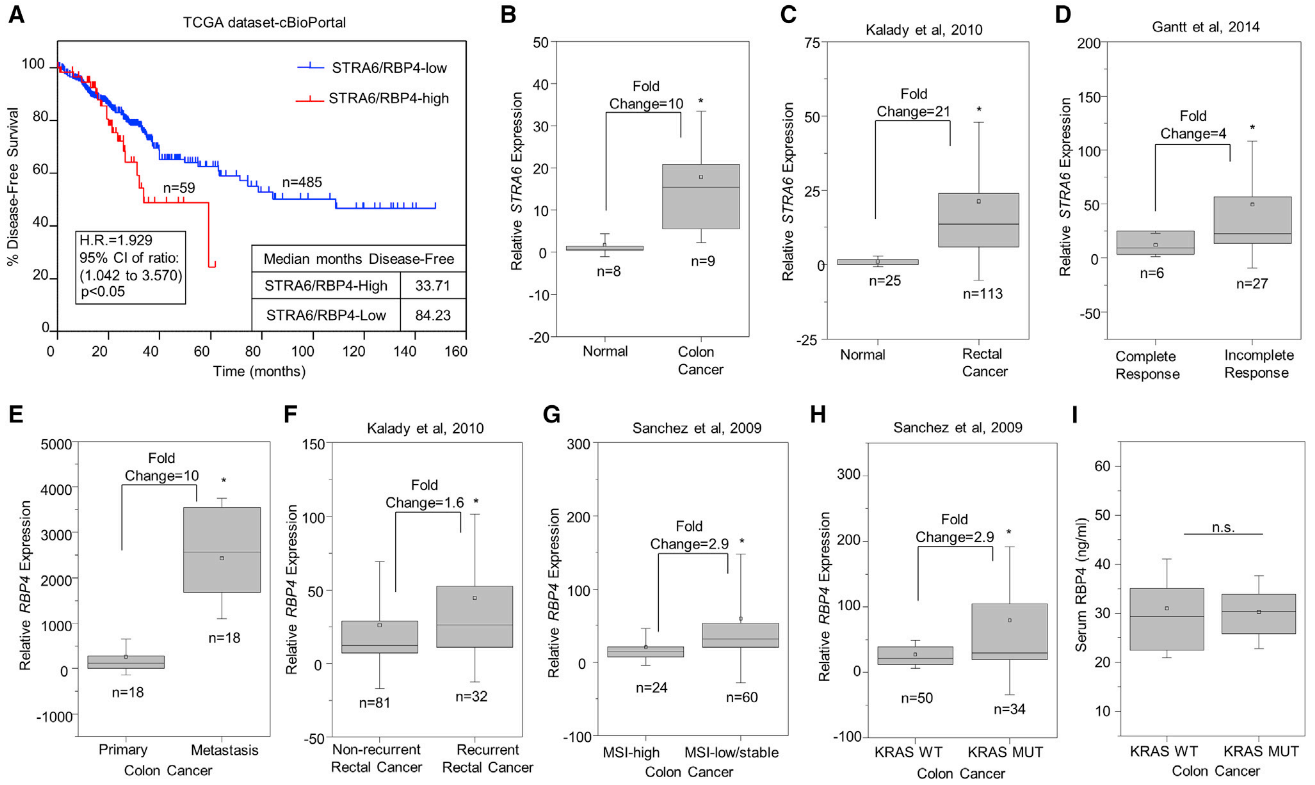

Figure 1. STRA6 and RBP4 Are Upregulated in Colorectal Cancer Patients

(A) Kaplan-Meier plot showing differences in disease-free survival percentages between colorectal cancer patients with high or low expression of STRA6 or RBP4. TCGA dataset, available through cBioPortal, was used. H.R., hazard ratio; CI, confidence interval.
(B and C) Levels of STRA6 expression in normal versus adenocarcinoma samples (B) and in normal versus rectal cancer patients (C).
(D) STRA6 mRNA levels in samples from patients showing complete or partial pathological response to chemoradiation.
(E) Data analysis showing levels of RBP4 mRNA in matched samples from primary and liver metastasis of colon cancer.
(F) Levels of RBP4 mRNA in rectal cancer patient samples grouped by 3-year recurrence.
(G and H) Levels of RBP4 mRNA in colon tumors with high versus low/stable microsatellite instability (MSI) (G) and in tumors with *KRAS* mutation (MUT) versus the wild-type (WT) (H).
(I) RBP4 levels measured in serum of KRAS WT (n = 16) and KRAS mutant (n = 14) patients.
Boxes represent the sample range and whiskers are 1 SD from the mean. Squares within the boxes represent mean values. $^*p < 0.05$; n.s., not significant

STRA6 or RBP4 were stably downregulated (Figures 2A–2C). Knockdown of STRA6 or RBP4 reduced the number of viable cells over time (Figure 2D). To test whether apoptotic properties were affected we treated SW480 cells with etoposide, a DNA-damaging agent. Etoposide treatment (72 hr) induced the cleavage of the apoptotic marker caspase-3 in control cells (Figure 2E). Knockdown of STRA6 or RBP4 increased the levels of cleaved caspase-3 compared with control cells stably expressing non-target shRNA (Figure 2E). The main characteristics of CSCs are their ability to proliferate indefinitely, reduce apoptotic rate, and self-renew (Reya et al., 2001). Our data so far demonstrate that both STRA6 and RBP4 affect cell proliferation and apoptosis, and therefore we next aimed to examine their effect on self-renewal. Analysis of the rectal cancer dataset showed upregulation of stemness markers, NANOG and LGR5 (Figure S2A). Hence, we investigated the effect of this pathway on the expression of core transcription factor machinery that regulates pluripotency. NANOG and SOX2 are key regulators of stem cell signature in embryonic (Niwa, 2007) as well as CSCs (Ben-Porath et al., 2008; Saigusa et al., 2009; Vaiopoulos et al., 2012). Knockdown of STRA6 or RBP4 in SW480 colon carcinoma cells decreased the levels of NANOG and SOX2 (Figures 2F and 2G). This effect was accompanied by a decrease in phosphorylated STAT3 levels (Figure S2B). Although STRA6 has a known role in intracellular transport of vitamin A in some tissues, ablation of STRA6 is established to have no effect on the levels of retinol or its oxidized product, retinoic acid, in most tissues (Berry et al., 2013). We verified that

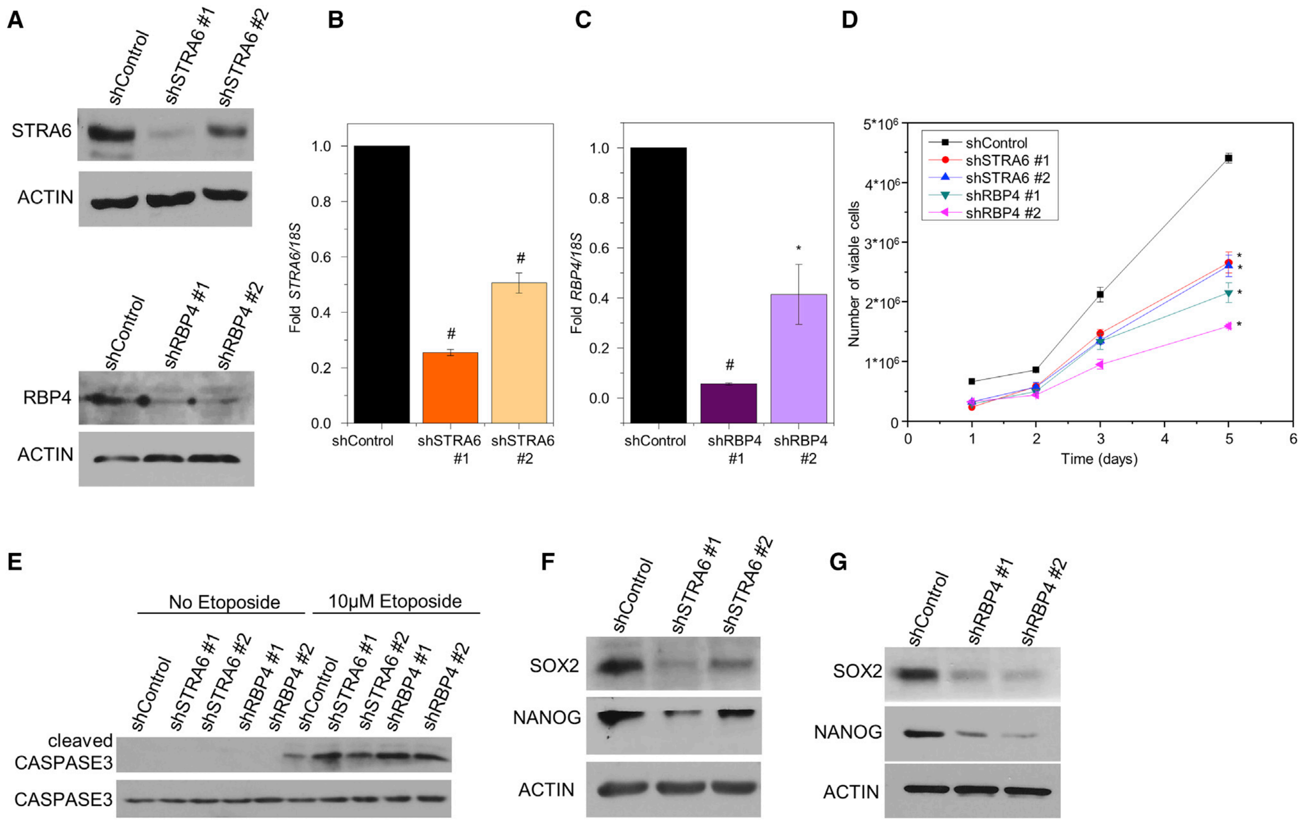

Figure 2. RBP4-STRA6 Pathway Is Necessary for Expression of CSC Markers

(A) Cell lysates from SW480 cells transfected with non-target, STRA6, or RBP4 shRNA were run on SDS-PAGE and immunoblotted with indicated antibodies.

(B and C) mRNA levels of *STRA6* (B) and *RBP4* (C) in the indicated SW480 stable lines. Data are presented as mean ± SD from n = 3 independent experiments *$p < 0.05$; $^{\#}p < 0.01$.

(D) Viability of SW480 cells stably expressing control, STRA6, or RBP4 shRNAs measured in triplicates for 5 days using trypan blue and Countess II FL. Data are presented as mean ± SE. *$p < 0.05$, significant difference between control and individual cell lines at each time point.

(E) Representative immunoblots of total and cleaved caspase-3 on cells in (A) treated with 10 μM etoposide for 72 hr.

(F and G) Representative immunoblots from three independent experiments showing levels of SOX2 and NANOG in SW480 stable lines. Cells were grown in delipidated medium for 18 hr before harvesting. Actin was used as loading control.

knockdown of STRA6 or RBP4 does not affect the levels of an endogenous target of retinoic acid, RARβ, in SW480 cells (Figure S2C). In addition, by using a Retinoic Acid Response Element (RARE)-luciferase reporter, we confirmed that knockdown of STRA6 or RBP4 in SW480 cells did not alter retinoic acid-dependent signaling (Figure S2D). Together, these data suggests that the RBP4-STRA6 pathway imparts pro-survival properties and is necessary for maintaining the expression of core stem cell transcription factors in SW480 cells.

The RBP4-STRA6 Pathway Is Necessary for Xenograft Growth and Survival

We have previously shown that knockdown of STRA6 in SW480 cells decreases tumor growth in a xenograft model (Berry et al., 2014). This was also accompanied by a delay in tumor initiation (Berry et al., 2014). To assess the role of RBP4 in tumor progression, we subcutaneously injected the SW480 line, in which RBP4 was stably downregulated using shRNA, into athymic nude mice. Decreasing RBP4 levels slowed the kinetics of tumor progression (Figure 3A). RBP4 knockdown caused a more than 3-fold reduction in tumor volume (Figures 3B and 3C) and significantly attenuated tumor initiation as indicated by the formation of only two tumors out of eight injected animals, unlike the control in which every injection resulted in tumor formation (Figure 3D). We examined the effect on stemness markers by analyzing the tumors resected at the endpoint of the experiment. As expected, RBP4 expression was reduced in knockdown tumors (Figure 3E). SOX2 and

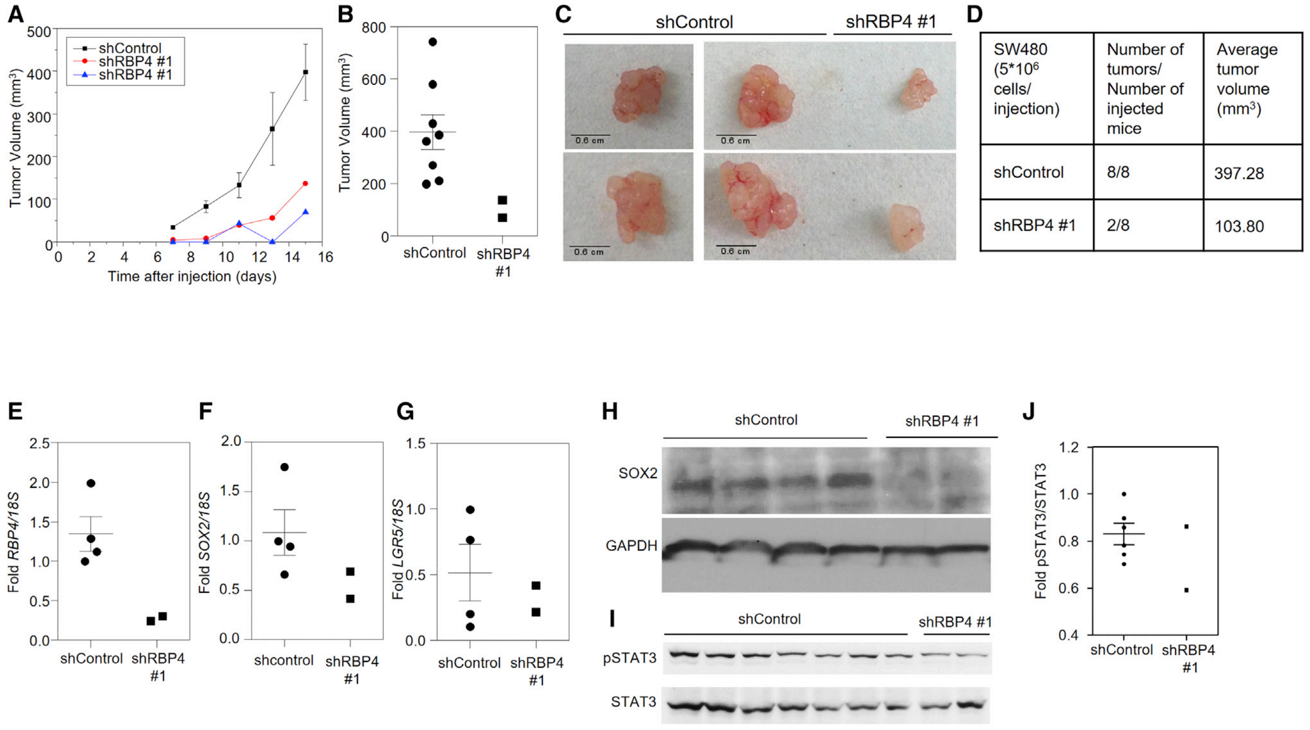

Figure 3. RBP4 Drives Tumor Survival of SW480 Cells

(A) Tumor growth in athymic NCr mice injected with SW480 cells (5×10^6) stably expressing non-target or RBP4 shRNA. Data for control tumors are mean ± SE (n = 8 tumors).

(B) Tumor volumes of individual tumor at 15 days after injection are plotted. Mean (n = 8 tumors) and SE for the control are shown.

(C) Representative tumors are shown that originate from SW480 cells stably expressing control or RBP4 shRNA.

(D) Average tumor volume at experimental endpoint and number of tumors initiated in each group are shown in the table.

(E–G) Levels of RBP4 (E), SOX2 (F), and LGR5 (G) mRNAs in SW480 tumors. Mean and SE for the control samples (n = 4 tumors) are shown.

(H) Immunoblots showing levels of SOX2 and GAPDH.

(I) Immunoblot showing levels of p-STAT3 and total STAT3 in SW480.

(J) Immunoblots in (I) were quantified using ImageJ software, and fold changes are presented. Mean and SEs for the control samples (n = 4 tumors) are shown.

LGR5 expression were trending toward a decrease in RBP4 knockdown tumors (Figures 3F and 3G). Consistent with gene expression, SOX2 protein levels were also reduced in the shRBP4 tumors (Figure 3H). Phosphorylated STAT3 levels were also trending toward a decrease in shRBP4 tumors (Figures 3I and 3J). The effect of RBP4 presented above, together with the previously shown effect of STRA6 on tumor progression (Berry et al., 2014), establish a role for the RBP4-STRA6 pathway in tumor initiation and suggest a potential role in the maintenance of colon CSCs.

STRA6 and RBP4 Regulate Colon CSC Maintenance

To test the effect of STRA6 and RBP4 on colon CSC maintenance, we performed an *in vitro* tumorsphere formation assay. Knockdown of STRA6 or RBP4 abrogated the ability of SW480 cells to initiate sphere formation (Figure 4A). To quantitatively assess differences in sphere-initiation ability due to changes in stem cell fraction, we performed a limiting dilution assay (LDA), a collective measure of proliferation, survival, and self-renewal. LDA showed that the sphere-initiating cell frequency was decreased upon knockdown of STRA6 (2.4- to 3.1-fold) or RBP4 (3.7- to 4.8-fold), indicating reduced stem cell maintenance (Figures 4B and S3A). To assess whether tumor-initiation efficiency of CSCs is affected, we performed an *in vivo* LDA whereby limiting dilution series of cells were injected into NOD-SCID IL2Rgamma (NSG) mice. Downregulating STRA6 or RBP4 reduced the tumor-initiating cell frequency by ~4.5-fold (Figures 4C and S3B). An *in vitro* secondary LDA from cells dissociated from control or STRA6 knockdown tumors showed a sustained decrease in sphere re-initiation frequency (Figure S3C). CD44 staining was used to enrich for CSCs present in the control tumors. Similarly to the stem cell marker LGR5, STRA6 and RBP4 expression levels were higher in the CSC fraction compared with the

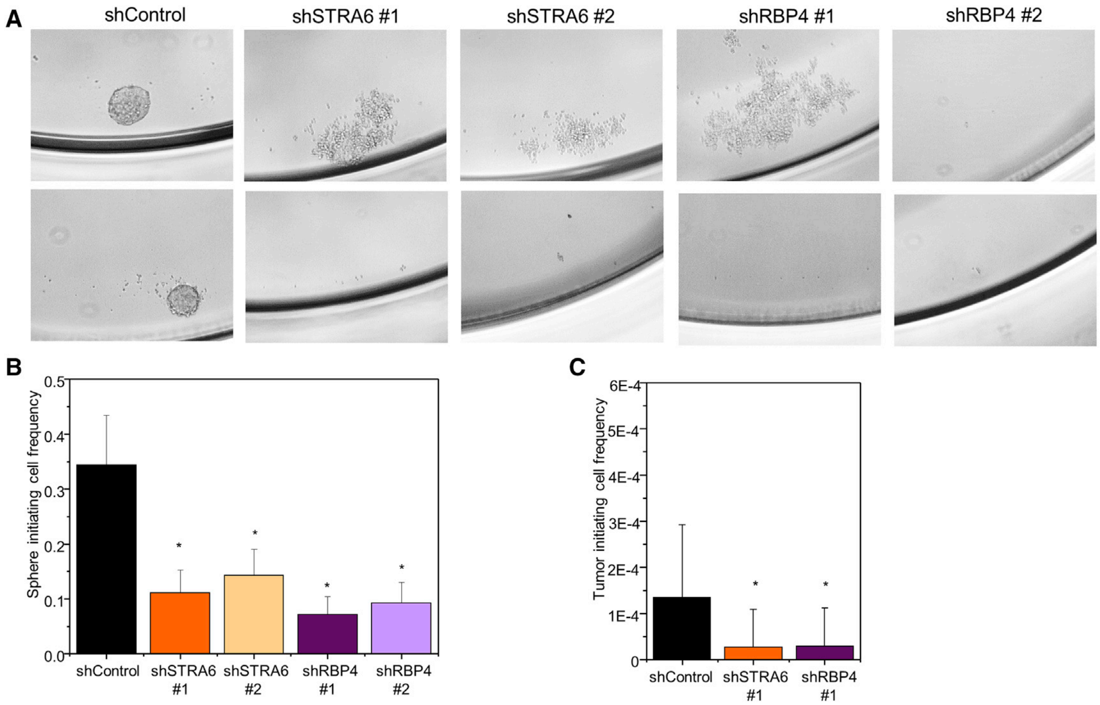

SW480	Sphere initiating-cell frequency		Fold Change	p value
	Estimate	95% Confidence interval		
shControl	1/2.91	(1/2.3-1/3.73)		
shSTRA6 #1	1/9.03	(1/6.56-1/12.5)	3.1	p<0.05
shSTRA6 #2	1/7.03	(1/5.25-1/9.49)	2.42	p<0.05
shRBP4 #1	1/14.03	(1/9.6-1/20.62)	4.82	p<0.05
shRBP4 #2	1/10.78	(1/7.67-1/15.26)	3.7	p<0.05

SW480	Tumor initiating-cell frequency		Fold Change	p value
	Estimate	95% Confidence Interval		
shControl	1/729	(1/3424-1/15990)		
shSTRA6 #1	1/36019	(1/9179-1/141342)	4.86	p<0.05
shRBP4 #1	1/33579	(1/8947-1/126033)	4.53	p<0.05

Figure 4. STRA6 and RBP4 Maintain CSC Frequency

(A) Low-magnification images of SW480 cells grown as non-adherent spheres. Loss of sphere formation ability upon STRA6 or RBP4 knockdown is shown.

(B) (Upper panel) Sphere-initiating cell frequency from an *in vitro* limiting dilution assay was calculated using ELDA software. A representative frequency estimate calculated from 24 biological replicates of each cell dose is shown. Error bars represent 95% confidence intervals. *$p < 0.05$. (Lower panel) Stem cell frequency estimates within confidence intervals and fold-change differences between control and designated lines.

(C) (Upper panel) SW480 cells stably transfected with STRA6 or RBP4 shRNA were injected at limiting doses into NOD-SCID gamma mice ($n = 5$–6 for each dose) and the ability to initiate tumor formation was evaluated. Plots show tumor-initiating cell frequency calculated using ELDA software. Error bars represent 95% confidence intervals.*$p < 0.05$. (Lower panel) Stem cell frequency estimates within confidence intervals and fold changes between control and indicated lines.

non-CSC fraction (Figure S3D). We extended this analysis further to a metastatic colon cancer cell line derived from a patient-derived xenograft (PDX 656). We verified that CD44 can be used as a marker to enrich for CSCs in the PDX line by performing an LDA. Indeed, the CD44-enriched population ($CD44^+$) showed higher sphere-initiating cell frequency compared with the $CD44^-$ fraction (Figure S4A), indicating a $CD44^+$ population enriched with CSCs. RNA analysis of $CD44^-$ versus $CD44^+$ populations showed that STRA6 expression was higher in the CSC fraction together with a moderate enrichment of NANOG (Figure S3E). LGR5 and RBP4 expression were not different between the two cell populations (Figure S3E). Together, these results suggest that the RBP4-STRA6 pathway is necessary for maintenance of the colon CSC pool.

STRA6 and RBP4 Regulate Stemness in a Patient-Derived Xenograft

To examine the contribution of the RBP4-STRA6 pathway to CSC maintenance in human specimens, we downregulated STRA6 and RBP4 using shRNAs in a PDX cell line

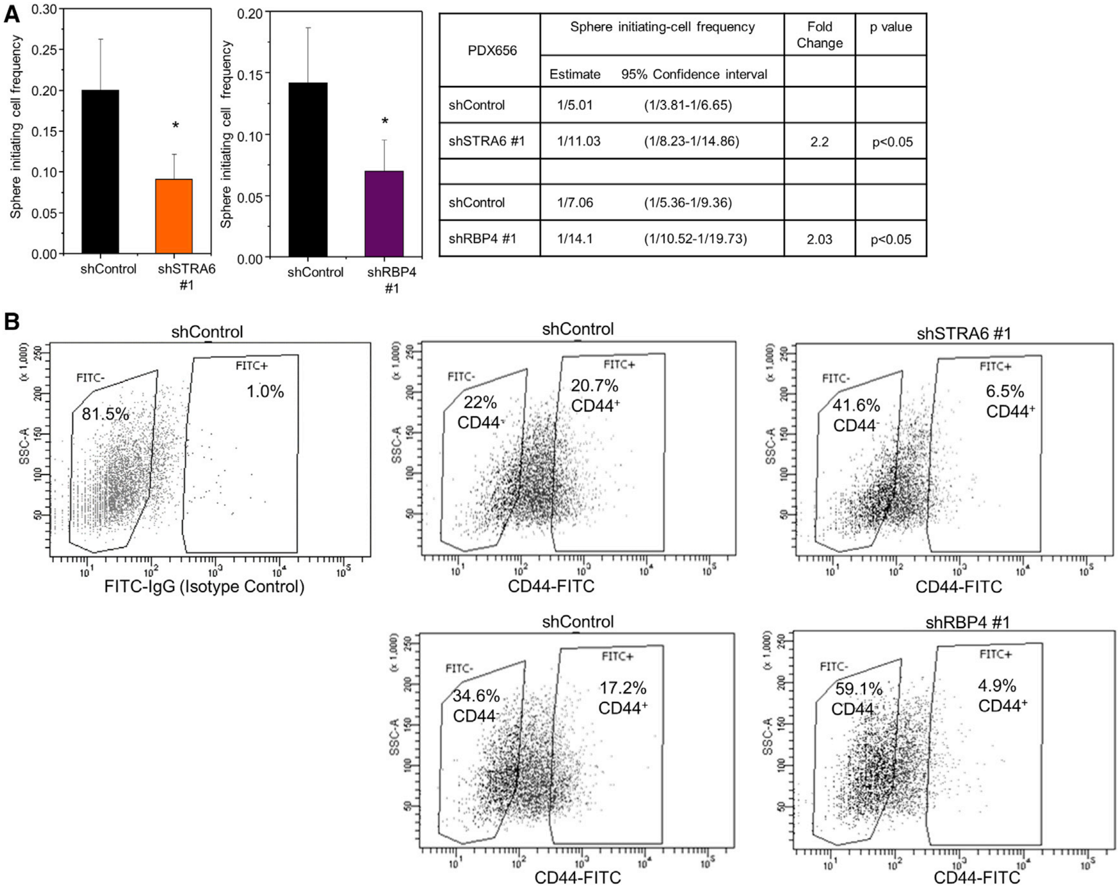

PDX656	Sphere initiating-cell frequency: Estimate	Sphere initiating-cell frequency: 95% Confidence interval	Fold Change	p value
shControl	1/5.01	(1/3.81-1/6.65)		
shSTRA6 #1	1/11.03	(1/8.23-1/14.86)	2.2	p<0.05
shControl	1/7.06	(1/5.36-1/9.36)		
shRBP4 #1	1/14.1	(1/10.52-1/19.73)	2.03	p<0.05

Figure 5. RBP4-STRA6 Pathway Maintains CSC Frequency in a PDX Model

(A) Sphere-initiating cell frequency of PDX 656 cells stably transfected with control or STRA6 (left) or RBP4 (right) shRNAs. A representative frequency estimate calculated from 24 biological replicates of each cell dose is shown. Error bars represent 95% confidence intervals. $*p < 0.05$. Stem cell frequency estimates within confidence intervals and fold differences between control and designated lines are shown in the table on the far right.

(B) Distribution of CD44-negative and -positive cells upon stable knockdown of STRA6 (top) or RBP4 (bottom) in PDX 656. FITC-immunoglobulin G (IgG) isotype control was used to set up the gates. Percentage of cells in each fraction is shown.

(Figure S4B). Decreased expression of STRA6 or RBP4 resulted in reduced sphere-initiating cell frequency in a LDA *in vitro* (Figures 5A and S4C). Knockdown of STRA6 or RBP4 also caused a substantial decrease in the percentage of $CD44^+$ cells (Figure 5B). These data indicate a significant role for the RBP4-STRA6 pathway in CSC maintenance.

High-Fat Diet Induces STRA6-Dependent LGR5 Expression in a Colon Cancer Xenograft

A recent study reported that HFD-induced obesity can increase intestinal stem cells and may thereby affect colon cancer risk (Beyaz et al., 2016). Because of the established role of the RBP4-STRA6 pathway in diet-induced metabolic syndrome (Berry et al., 2013; Yang et al., 2005) and the results presented above on their role in CSC maintenance, we investigated whether the RBP4-STRA6 pathway could provide a link between HFD feeding and colon cancer stemness. To delineate the role of HFD and eliminate obesity as a confounding factor, we used athymic nude mice that are resistant to HFD-induced obesity (Montgomery et al., 2013). This provided an important tool to investigate the impact of HFD on stemness independent of obesity. Mice that were fed either normal chow or HFD for 15 weeks were then injected subcutaneously with SW480 cells stably expressing control (shGFP) or STRA6 shRNA, and tumor growth was monitored for 2 additional weeks while being maintained on the dietary regimen. No differences were found in food intake (Figure S5) or body weight of mice on the different diets (Figure 6A). However, serum levels of RBP4 were increased by high-fat feeding (Figure 6A, inset). In our recent publication, we showed that STRA6 knockdown delayed tumor initiation by 4 days and

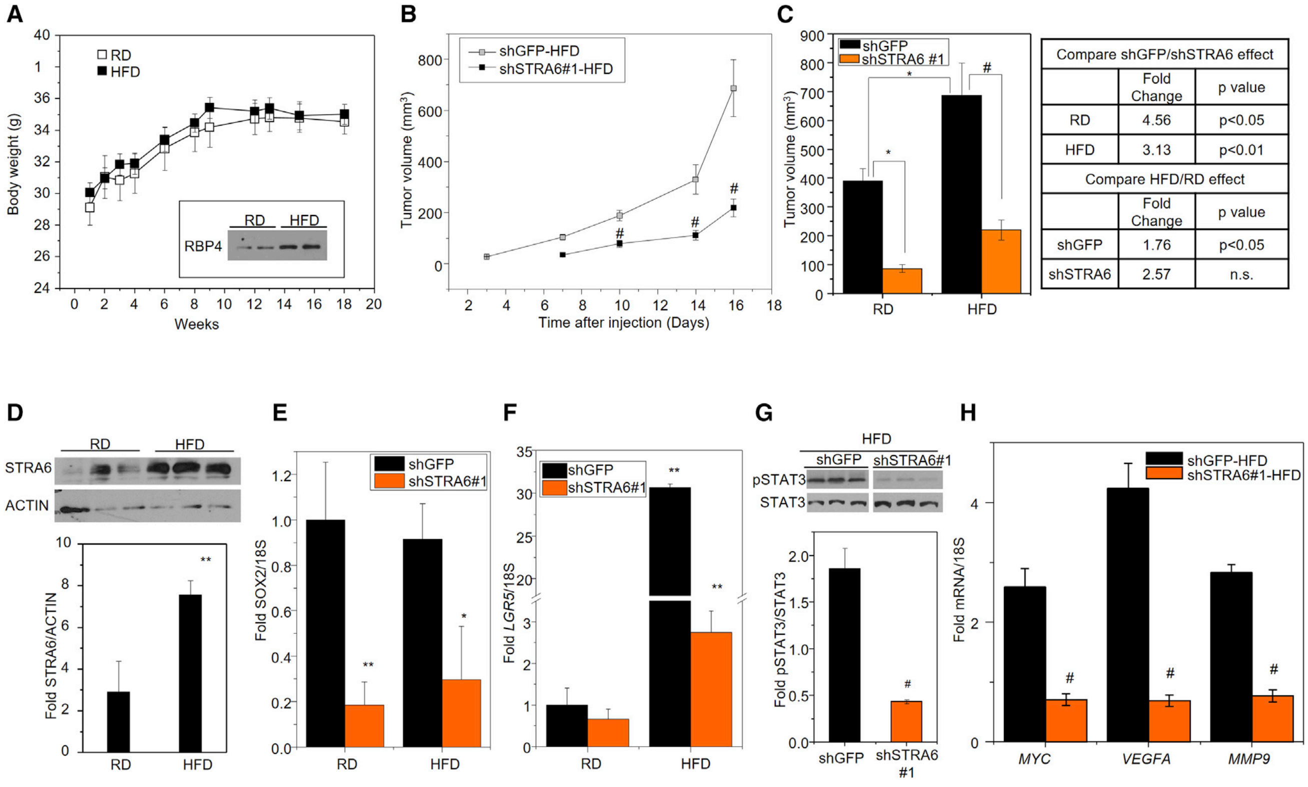

Figure 6. HFD Increases STRA6 and LGR5 in a Xenograft Model

(A) Body weight of NCr nude mice fed either a regular rodent chow (RD) or a high-fat/high-sucrose diet (HFD) for 18 weeks. Arrow indicates the time point (15 weeks) at which cells were injected. Inset: immunoblot of RBP4 in serum of RD- or HFD-fed mice after 15 weeks. Data are mean ± SEM (n = 10 mice).

(B) NCr male mice in (A) were injected with 5×10^6 SW480 cells stably expressing GFP shRNA or STRA6 shRNA after 15 weeks on HFD. Tumor growth at both injected sites was monitored twice a week. Data are mean ± SEM (n = 7–9 tumors). $^{\#}p < 0.01$

(C) Tumor volumes at endpoint of the experiment in (A). Data are mean ± SEM (n = 7–9 tumors). $^{*}p < 0.05$; $^{\#}p < 0.01$. Fold changes in tumor volume between different conditions and their statistical analysis is shown in the table on the right.

(D) (Top) Immunoblot for STRA6 in tumors at endpoint in (A). (Bottom) Quantification of the immunoblot. Data are mean ± SEM (n = 3 tumors). $^{**}p < 0.05$.

(E and F) Expression levels of *SOX2* (E) and *LGR5* (F) in tumors at endpoint in (B). Data are mean ± SEM (n = 3–5 tumors). $^{*}p < 0.01$; $^{**}p < 0.05$.

(G) Immunoblot for pSTAT3 in tumors arising from SW480 stable lines in mice fed an HFD (top) and the quantification of the immunoblot (bottom). Data are mean ± SEM (n = 3 tumors). $^{\#}p < 0.01$

(H) Expression levels of STAT3 target genes in the same tumors in (G) from mice fed an HFD. Data are mean ± SD (n = 3 tumors). $^{\#}p < 0.01$.

decreased tumor progression in mice fed normal chow (Berry et al., 2014). Here, we examine the effect of HFD on tumor growth in the presence and absence of STRA6. Knockdown of STRA6 significantly decreased tumor progression on an HFD (Figure 6B) and final tumor volumes were decreased on both regular rodent chow diet (RD) and HFD (Figure 6C). HFD caused a significantly increased tumor growth by about 1.8-fold in control tumors, and the tumor burden (Figure 6C) was also higher compared with mice fed normal chow (mean ± SE tumor volume 389.37 ± 42.81 [shControl-RD] versus 686.92 ± 112.07 [shControl-HFD]; $p < 0.05$). However, HFD did not induce a significant increase in tumor growth in shSTRA6 cells (tumor volume 85.37 ± 13.48 [shSTRA6-RD] versus 219.16 ± 35.08 [shSTRA6-HFD]; not significant) (Figure 6C). The HFD-mediated increase in tumor growth in control tumors was accompanied by at least a 2-fold increase in STRA6 levels (Figure 6D).

Reducing STRA6 levels delayed tumor initiation (Figure 6B) in addition to decreasing tumor progression. Analysis of the tumors showed that the delay in tumor initiation was associated with decreased expression of stem cell marker SOX2 (Figure 6E). Interestingly, HFD feeding caused a significant increase in the levels of LGR5 (Figure 6F).

Although LGR5 was only trending toward a decrease on RD, its marked upregulation on HFD feeding was significantly abrogated by downregulating STRA6 (Figure 6F). In addition, loss of STRA6 in HFD was accompanied by a decrease in phosphorylation of STAT3 (Figure 6G) and reduced expression of JAK-STAT target genes, VEGFA, MYC, and MMP9 (Figure 6H). These data indicate that HFD induces tumor progression together with the levels of STRA6 and LGR5, and this effect is at least partly regulated by the RBP4-STRA6 pathway.

DISCUSSION

Proteins involved in vitamin A signaling and retinoic acid metabolism including STRA6 (Berry et al., 2014; Szeto et al., 2001), RBP4 (Berry et al., 2014), and many intracellular lipid binding proteins (Levi et al., 2013; Manor et al., 2003) have been implicated in tumorigenesis of several cancers. However, not much is known about a possible role of these pathways in CSC maintenance. Here we show a role of the RBP4-STRA6 pathway in regulating and maintaining colon CSCs. Cancer stem cell properties are thought to contribute to tumor recurrence, therapeutic resistance, and metastasis in colon cancer (Vaiopoulos et al., 2012). Indeed, our data show that STRA6 and RBP4 are upregulated in colorectal cancers compared with normal colon, and also in more advanced disease with worse prognosis. Furthermore, we establish a connection between high fat intake and colon cancer risk via the RBP4-STRA6 pathway.

Our data clearly indicate that RBP4-STRA6 pathway is necessary for the optimal expression of stem cell markers such as NANOG, SOX2, and LGR5, and thereby for maintaining the colon CSC pool. One possible mechanism by which the RBP4-STRA6 pathway could regulate stemness is via STAT3 activation. STAT3 is known to play a role in cancer progression and stemness (Bromberg et al., 1999). Activated STAT3 can bind to regulatory elements on the promoters of the core pluripotency transcription factors NANOG, SOX2, and OCT4 in embryonic stem cells (Do et al., 2013; Foshay and Gallicano, 2008). We have established that activation of the RBP4-STRA6 pathway results in phosphorylation of STAT3 (Berry et al., 2011, 2012, 2013, 2014; Berry and Noy, 2012), suggesting that STAT3 activation may directly regulate expression of these targets in CSCs.

A recent report on the structural determination of STRA6 revealed a new interacting partner, calmodulin (CaM) (Chen et al., 2016). It is interesting to note that calmodulin and CaM-dependent pathways have established roles in differentiation of stem cells and decreasing CSC maintenance in a glioblastoma model (Berchtold and Villalobo, 2014; Schraivogel et al., 2011). An interesting possibility could be that the RBP4-STRA6 pathway is maintained at a resting state while bound to CaM resulting in a differentiated program, while its aberrant activation during colon cancer pathologies lead to increased stem cell properties and tumor progression.

Furthermore, our work reveals a role for the RBP4-STRA6 pathway in regulating HFD-induced expression of LGR5 independent of obesity and, subsequently, the increased kinetics of tumor progression. A latest report shows a potential mechanism by which HFD upregulates LGR5 and stemness by activating the Wnt pathway (Beyaz et al., 2016). Interestingly, Wnt activity has also been previously shown to increase STRA6 expression together with retinoic acid (Szeto et al., 2001). These studies together with the data presented here suggest a possible diet-induced crosstalk between the Wnt pathway and the RBP4-STRA6 pathway in colon carcinogenesis.

Our results show that RBP4 expression in tumors and not serum RBP4 levels were associated with aggressive forms of colon cancer (Figures 1H and 1I). This is in agreement with our previous study showing that SW480 cells secreted RBP4 into culture medium, activating STRA6 signaling in an autocrine manner (Berry et al., 2014). These results indicate that the intratumoral concentration and secretion of RBP4 into the tumor microenvironment seem to mediate CSC properties. However, an additional STRA6-independent role for RBP4 cannot be completely dismissed. In fact, RBP4 has been shown to play a role in TLR4-mediated inflammatory signaling and insulin resistance independent of STRA6 (Norseen et al., 2012). The exact contributions of intracellular and serum RBP4 to colon CSC maintenance remains to be clarified.

EXPERIMENTAL PROCEDURES

Reagents

Non-target shRNA control (SHC002) and RBP4shRNAs (TRCN0000060038 [#1] and TRCN0000060039 [#2]) were purchased from Sigma. STRA6shRNAs (TRCN0000128799 [#2] and TRCN0000129158 [#1]) and EGFP (RHS4459) were from Open Biosystems. Epidermal growth factor (EGF) was from Stem Cell Technologies and basic fibroblast growth factor (FGF) was from Peprotech. Etoposide, insulin, and heparin were purchased from Sigma. Viable cells were counted after trypan blue staining using Countess II FL (Life Technologies). Human RBP4 in serum samples of patients was quantified using an RBP4 Quantikine ELISA kit (R&D Systems) following the manufacturer's protocols.

Cell Culture and Transfection

Cell lines were obtained from ATCC and periodically checked for mycoplasma contamination using a mycoplasma detection kit (Sigma). SW480 colon adenocarcinoma cells were maintained as

adherent cultures in McCoy's 5A medium supplemented with 10% fetal bovine serum (FBS) and penicillin-streptomycin (Corning). 293T cells were cultured in DMEM with 10% FBS. Transient transfections were performed using Polyfect (Qiagen). Stable knockdowns were obtained by lentiviral shRNA infections. Lentiviral packaging was achieved in 293T cells by co-transfecting with packaging (pCMV) and envelope (pMD2.G) plasmids. Puromycin was used to select for stable cell lines.

Patient-Derived Xenograft Cell Line

A patient-derived xenograft cell line (PDX 656) from brain metastasis of colon cancer (patient ID: 656) has been previously described (Lotti et al., 2013) and was maintained by passaging in NSG mice. Authenticity was verified by comparing the histology with the parental tumor. PDX 656 cells were grown as non-adherent cultures in DMEM/F12 medium supplemented with 1× B27 supplements (Life Technologies), insulin, 20 ng/mL EGF, 10 ng/mL FGF, and 4 μg/mL heparin.

Flow Cytometry

All flow-cytometry analyses were performed at the Lerner Research Institute Flow Cytometry core. Cells were processed in flow buffer (0.5% BSA, 2 mM EDTA in PBS) and DAPI-negative viable cells were sorted on a BD FACSAria II. CD44-fluorescein isothiocyanate (FITC) (BD Biosciences, #555478) antibody was used to stain CD44. Enrichment of CD44-positive cells was performed by magnetic labeling of the cells with CD44 microbeads (Miltenyi Biotech, #130-095-194) followed by separation on a MACS column (Miltenyi Biotech).

Western Blotting

Cells and tumors were lysed in RIPA buffer (150 mM NaCl, 10 mM Tris [pH 7.5], 0.1% SDS, 1% Triton X-100, 1% sodium deoxycholate, 5 mM EDTA, and Halt protease inhibitor cocktail [Pierce]) and total protein was quantified using Bio-Rad protein assay reagent. An equal amount of protein was loaded on 10% or 15% SDS-PAGE gels and immunoblotted. STRA6 antibody was generated in the Lerner Research Institute Molecular Biotechnology core as previously described (Berry et al., 2013; Bouillet et al., 1997). RBP4 antibody was purchased from Atlas Antibodies (HPA001641) and serum RBP4 was blotted using antibody from Dako (#A0040). Antibodies to NANOG (#4903), SOX2 (#3579), STAT3 (#4904), and phospho-STAT3 (Y705) (#9145) were from Cell Signaling Technology. RARβ (SC-552), βActin (SC-47778), and GAPDH (SC-32233) antibodies were from Santa Cruz Biotechnology.

RNA Isolation and RT-PCR Analysis

RNA isolation was carried out using RNAZol reagent (MRC). RNA was reverse transcribed and cDNA was generated using the Ecodry RNA-tocDNA kit (Clontech). Expression analysis was performed using TaqMan Fast Universal PCR Master Mix in a StepOnePlus Real-Time PCR system (Thermo Fisher) with Taqman probes (Thermo Fisher): 18s (4352930) rRNA, STRA6 (Hs00980261_g1), RBP4 (Hs00924047_m1), MMP9 (Hs00957562_m1), MYC (Hs99999003_m1), VEGFA (Hs00900055_m1), NANOG (Hs04399610_g1), SOX2 (Hs01053049_s1), and LGR5 (Hs00969422_m1).

Mouse Experiments

Six-week-old NCr/nude male mice were injected with 5 × 10^6 SW480 cells stably expressing control SHC002 (shControl) into the right flank and SW480 cells stably expressing shRBP4 #1 (TRCN0000060038) into the left flank. Tumor growth was monitored using a vernier caliper every 2 days. Tumor volumes were calculated using (length × $\text{width}^2/2$).

In Vivo Limiting Dilution Assay

SW480 cells stably transfected with control (shControl), STRA6 (shSTRA6 #1), or RBP4 (shRBP4 #1) shRNAs were used. Limiting doses of viable cells were injected subcutaneously into 6-week-old NOD-SCID gamma (NSG) male mice. For control and STRA6 knockdown, 10,000, 5,000, and 1,500 cells each were used (n = 5). Limiting dilution assay with RBP4 knockdown was performed with 250,000 (n = 5), 15,000 (n = 5) and 1,500 (n = 6) cells. Six weeks after injection, mice were scored for the presence of palpable tumors. The dose of cells injected to the fraction of mice without tumors was plotted and slope of the graph was used to estimate the fraction of stem cells using extreme limiting dilution analysis (ELDA) software. The term "tumor-initiating cell frequency" is used to define the estimated fraction of stem cells based on their ability to initiate tumor formation *in vivo*. Tumor-initiating cell frequency was calculated using ELDA software (http://bioinf.wehi.edu.au/software/elda/) (Hu and Smyth, 2009).

High-Fat Diet Studies

Six-week-old NCr/nude male mice were separated into two groups and were fed either a regular rodent chow (RD) or a high-fat/high-sucrose diet (D12331) for 18 weeks. Body weight was recorded every week. After 15 weeks, plasma RBP4 levels were verified to be increased upon HFD feeding, and mice were injected with 5 × 10^6 SW480 cells stably expressing GFP shRNA into the left flank and SW480 cells stably expressing STRA6 shRNA #1 into the right flank. Tumor size was measured using a vernier caliper at both injection sites twice a week. Tumor volumes were calculated using (length × $\text{width}^2/2$).

Sphere Formation Assay

SW480 cells were grown as spheres in DMEM/F12 supplemented with B27 (Life Technologies), 20 ng/mL EGF, and 20 ng/mL FGF. Viable cells were sorted at limiting doses (1, 2, 5, and 10 cells) into 96-well non-tissue-culture-treated plates and spheres were scored 12 days after growth at 37°C and 5% CO_2. The dose of cells to the fraction of wells without spheres was plotted, and the slope of the graph was used to estimate the fraction of stem cells using ELDA software. The term "sphere-initiating cell frequency" is used to define the estimated fraction of stem cells based on their ability to initiate sphere formation in culture.

Patient Data and Microarray Analysis

All human tissues were acquired from primary human colorectal tumor patient specimens according to human experimental guidelines. Human protocols were approved by the Institutional Review Board of the Cleveland Clinic Foundation (IRB 4134). Frozen tissue samples from nine normal and nine tumor specimens used in Figure 1B were cut using a Leica CM1850 cryostat in 10-μm sections,

and RNA was extracted using the RNAqueous Total RNA Isolation kit (AM1912, Ambion).

We used previously published microarray datasets on patient samples to examine the expression levels of STRA6 and RBP4. In brief, tissue samples from normal (n = 25) and rectal cancer patients (n = 113) were used to isolate RNA and data were generated using the Illumina platform, described in detail in Hogan et al. (2015b). Samples from 33 rectal cancer patients who underwent standard neoadjuvant chemoradiation therapy with 5,040 Gy radiation and 5-fluorouracil were included to evaluate therapy response. Tumor samples resected during curative proctectomy were stratified as pathological complete responders (complete response) or partial or incomplete responders (Incomplete response). RNA isolation followed by microarray analysis using Illumina Platform human-6 v2 containing 48,701 probes was performed for all the samples and have been previously described (Gantt et al., 2014).

For expression analysis of RBP4, matched primary colon and liver metastatic samples (n = 18) were used for RNA isolation and microarray as described above. Furthermore, early-stage rectal cancer patients (n = 113) treated exclusively by surgery were followed up for a 3-year period and classified based on tumor relapse. Microarray data were generated from recurrent and non-recurrent rectal cancer patients (n = 113) using an Illumina-based Sentrix Human-6 Expression Beadchip described in detail previously (Kalady et al., 2010). Another dataset comprising of stage II and III colon adenocarcinoma patient samples (n = 84) was used to isolate genomic DNA and RNA. Genomic DNA was tested for ten different microsatellite markers and stratified as microsatellite instability-high (>30% of markers) or microsatellite instability-low/stable ($\leq$30% of markers). A detailed description of the panel of microsatellite markers used and the analysis are described in Sanchez et al. (2009). KRAS-mutation status of tumor samples was identified based on PCR amplification of KRAS region followed by sequencing (Sanchez et al., 2009). Microarray on these RNA samples was performed using Illumina Human-6 Expression v2 Beadchip as detailed in Hogan et al. (2015a). STRA6 and RBP4 expression were compared between the aforementioned groups.

Database and Statistical Analysis

Statistical analysis was performed using Student's t test. When more than two groups were analyzed, one-way ANOVA was used to calculate statistical significance. Pearson's coefficient was used to analyze correlation of gene expression using GraphPad Prism 5. Survival data were downloaded from cBioPortal (http://www.cbioportal.org/) (Cerami et al., 2012; Gao et al., 2013) and Kaplan-Maier analysis was performed using GraphPad Prism 5. Patients with STRA6 or RBP4 expression greater than 1 SD of mean of all patients were classified as the high-expression group.

RARE Transactivation Assay

SW480 cells were transfected with RARα, RARE-driven luciferase reporter, and β-galactosidase in triplicates. Eighteen hours after transfection, cells were treated with serum-free medium containing 1 μM retinoic acid for 18 hr. Cells were then lysed, and luciferase activity was measured by following the manufacturer's protocol (Promega) and normalized to β-galactosidase to control for transfection efficiency.

SUPPLEMENTAL INFORMATION

Supplemental Information includes five figures and can be found with this article online at http://dx.doi.org/10.1016/j.stemcr.2017.06.002.

AUTHOR CONTRIBUTIONS

S.K. and N.N. conceived the idea, and N.N. supervised the study. S.K. designed and performed experiments, analyzed data, and wrote the manuscript. L.L. designed and carried out experiments and helped with data analysis and editing of the manuscript. G.K. performed the microarray analyses, and J.D. provided technical support under the supervision of M.F.K. J.D.L. and O.R. provided comments on the manuscript and helped with drafting the revisions.

ACKNOWLEDGMENTS

This work was supported by NIH grant R01DK088969 awarded to N.N.

Received: November 9, 2016
Revised: May 31, 2017
Accepted: June 1, 2017
Published: July 6, 2017

REFERENCES

Abola, M.V., Thompson, C.L., Chen, Z., Chak, A., Berger, N.A., Kirwan, J.P., and Li, L. (2015). Serum levels of retinol-binding protein 4 and risk of colon adenoma. Endocr. Relat. Cancer *22*, L1–L4.

Ben-Porath, I., Thomson, M.W., Carey, V.J., Ge, R., Bell, G.W., Regev, A., and Weinberg, R.A. (2008). An embryonic stem cell-like gene expression signature in poorly differentiated aggressive human tumors. Nat. Genet. *40*, 499–507.

Berchtold, M.W., and Villalobo, A. (2014). The many faces of calmodulin in cell proliferation, programmed cell death, autophagy, and cancer. Biochim. Biophys. Acta *1843*, 398–435.

Berry, D.C., and Noy, N. (2012). Signaling by vitamin A and retinol-binding protein in regulation of insulin responses and lipid homeostasis. Biochim. Biophys. Acta *1821*, 168–176.

Berry, D.C., Jin, H., Majumdar, A., and Noy, N. (2011). Signaling by vitamin A and retinol-binding protein regulates gene expression to inhibit insulin responses. Proc. Natl. Acad. Sci. USA *108*, 4340–4345.

Berry, D.C., O'Byrne, S.M., Vreeland, A.C., Blaner, W.S., and Noy, N. (2012). Cross talk between signaling and vitamin A transport by the retinol-binding protein receptor STRA6. Mol. Cell. Biol. *32*, 3164–3175.

Berry, D.C., Jacobs, H., Marwarha, G., Gely-Pernot, A., O'Byrne, S.M., DeSantis, D., Klopfenstein, M., Feret, B., Dennefeld, C., Blaner, W.S., et al. (2013). The STRA6 receptor is essential for retinol-binding protein-induced insulin resistance but not for maintaining vitamin A homeostasis in tissues other than the eye. J. Biol. Chem. *288*, 24528–24539.

Berry, D.C., Levi, L., and Noy, N. (2014). Holo-retinol-binding protein and its receptor STRA6 drive oncogenic transformation. Cancer Res. *74*, 6341–6351.

Beyaz, S., Mana, M.D., Roper, J., Kedrin, D., Saadatpour, A., Hong, S.J., Bauer-Rowe, K.E., Xifaras, M.E., Akkad, A., Arias, E., et al. (2016). High-fat diet enhances stemness and tumorigenicity of intestinal progenitors. Nature *531*, 53–58.

Bouillet, P., Sapin, V., Chazaud, C., Messaddeq, N., Decimo, D., Dolle, P., and Chambon, P. (1997). Developmental expression pattern of Stra6, a retinoic acid-responsive gene encoding a new type of membrane protein. Mech. Dev. *63*, 173–186.

Bromberg, J.F., Wrzeszczynska, M.H., Devgan, G., Zhao, Y., Pestell, R.G., Albanese, C., and Darnell, J.E., Jr. (1999). Stat3 as an oncogene. Cell *98*, 295–303.

Cerami, E., Gao, J., Dogrusoz, U., Gross, B.E., Sumer, S.O., Aksoy, B.A., Jacobsen, A., Byrne, C.J., Heuer, M.L., Larsson, E., et al. (2012). The cBio cancer genomics portal: an open platform for exploring multidimensional cancer genomics data. Cancer Discov. *2*, 401–404.

Chen, Y., Clarke, O.B., Kim, J., Stowe, S., Kim, Y.K., Assur, Z., Cavalier, M., Godoy-Ruiz, R., von Alpen, D.C., Manzini, C., et al. (2016). Structure of the STRA6 receptor for retinol uptake. Science *353*. http://dx.doi.org/10.1126/science.aad8266.

Colussi, D., Brandi, G., Bazzoli, F., and Ricciardiello, L. (2013). Molecular pathways involved in colorectal cancer: implications for disease behavior and prevention. Int. J. Mol. Sci. *14*, 16365–16385.

Dick, J.E. (2008). Stem cell concepts renew cancer research. Blood *112*, 4793–4807.

Do, D.V., Ueda, J., Messerschmidt, D.M., Lorthongpanich, C., Zhou, Y., Feng, B., Guo, G., Lin, P.J., Hossain, M.Z., Zhang, W., et al. (2013). A genetic and developmental pathway from STAT3 to the OCT4-NANOG circuit is essential for maintenance of ICM lineages *in vivo*. Genes Dev. *27*, 1378–1390.

Foshay, K.M., and Gallicano, G.I. (2008). Regulation of Sox2 by STAT3 initiates commitment to the neural precursor cell fate. Stem Cells Dev. *17*, 269–278.

Gantt, G.A., Chen, Y., Dejulius, K., Mace, A.G., Barnholtz-Sloan, J., and Kalady, M.F. (2014). Gene expression profile is associated with chemoradiation resistance in rectal cancer. Colorectal Dis. *16*, 57–66.

Gao, J., Aksoy, B.A., Dogrusoz, U., Dresdner, G., Gross, B., Sumer, S.O., Sun, Y., Jacobsen, A., Sinha, R., Larsson, E., et al. (2013). Integrative analysis of complex cancer genomics and clinical profiles using the cBioPortal. Sci. Signal. *6*, pl1.

Hogan, J., DeJulius, K., Liu, X., Coffey, J.C., and Kalady, M.F. (2015a). Transcriptional profiles underpin microsatellite status and associated features in colon cancer. Gene *570*, 36–43.

Hogan, J., Dejulius, K., Liu, X., Coffey, J.C., and Kalady, M.F. (2015b). Transcriptomic profiles differentiate normal rectal epithelium and adenocarcinoma. Int. Surg. *100*, 818–826.

Howlader, N., Noone, A., Krapcho, M., Miller, D., Bishop, K., Altekruse, S., Kosary, C., Yu, M., Ruhl, J., Tatalovich, Z., et al. (2016). SEER Cancer Statistics Review, 1975-2013 (National Cancer Institute), based on November 2015 SEER data submission, posted to the SEER website, April 2016. https://seer.cancer.gov/csr/1975_2013/

Hu, Y., and Smyth, G.K. (2009). ELDA: extreme limiting dilution analysis for comparing depleted and enriched populations in stem cell and other assays. J. Immunol. Methods *347*, 70–78.

Kalady, M.F., Dejulius, K., Church, J.M., Lavery, I.C., Fazio, V.W., and Ishwaran, H. (2010). Gene signature is associated with early stage rectal cancer recurrence. J. Am. Coll. Surg. *211*, 187–195.

Kawaguchi, R., Yu, J., Honda, J., Hu, J., Whitelegge, J., Ping, P., Wiita, P., Bok, D., and Sun, H. (2007). A membrane receptor for retinol binding protein mediates cellular uptake of vitamin A. Science *315*, 820–825.

Kreso, A., and Dick, J.E. (2014). Evolution of the cancer stem cell model. Cell Stem Cell *14*, 275–291.

Lao, V.V., and Grady, W.M. (2011). Epigenetics and colorectal cancer. Nat. Rev. Gastroenterol. Hepatol. *8*, 686–700.

Levi, L., Lobo, G., Doud, M.K., von Lintig, J., Seachrist, D., Tochtrop, G.P., and Noy, N. (2013). Genetic ablation of the fatty acid-binding protein FABP5 suppresses HER2-induced mammary tumorigenesis. Cancer Res. *73*, 4770–4780.

Lotti, F., Jarrar, A.M., Pai, R.K., Hitomi, M., Lathia, J., Mace, A., Gantt, G.A., Jr., Sukhdeo, K., DeVecchio, J., Vasanji, A., et al. (2013). Chemotherapy activates cancer-associated fibroblasts to maintain colorectal cancer-initiating cells by IL-17A. J. Exp. Med. *210*, 2851–2872.

Manor, D., Shmidt, E.N., Budhu, A., Flesken-Nikitin, A., Zgola, M., Page, R., Nikitin, A.Y., and Noy, N. (2003). Mammary carcinoma suppression by cellular retinoic acid binding protein-II. Cancer Res. *63*, 4426–4433.

Merlos-Suarez, A., Barriga, F.M., Jung, P., Iglesias, M., Cespedes, M.V., Rossell, D., Sevillano, M., Hernando-Momblona, X., da Silva-Diz, V., Munoz, P., et al. (2011). The intestinal stem cell signature identifies colorectal cancer stem cells and predicts disease relapse. Cell Stem Cell *8*, 511–524.

Montgomery, M.K., Hallahan, N.L., Brown, S.H., Liu, M., Mitchell, T.W., Cooney, G.J., and Turner, N. (2013). Mouse strain-dependent variation in obesity and glucose homeostasis in response to high-fat feeding. Diabetologia *56*, 1129–1139.

Newmark, H.L., Yang, K., Lipkin, M., Kopelovich, L., Liu, Y., Fan, K., and Shinozaki, H. (2001). A Western-style diet induces benign and malignant neoplasms in the colon of normal C57Bl/6 mice. Carcinogenesis *22*, 1871–1875.

Ning, Y., Wang, L., and Giovannucci, E.L. (2010). A quantitative analysis of body mass index and colorectal cancer: findings from 56 observational studies. Obes. Rev. *11*, 19–30.

Niwa, H. (2007). How is pluripotency determined and maintained? Development *134*, 635–646.

Norseen, J., Hosooka, T., Hammarstedt, A., Yore, M.M., Kant, S., Aryal, P., Kiernan, U.A., Phillips, D.A., Maruyama, H., Kraus, B.J., et al. (2012). Retinol-binding protein 4 inhibits insulin signaling in adipocytes by inducing proinflammatory cytokines in macrophages through a c-Jun N-terminal kinase- and toll-like receptor 4-dependent and retinol-independent mechanism. Mol. Cell. Biol. *32*, 2010–2019.

Noy, N. (2000). Retinoid-binding proteins: mediators of retinoid action. Biochem. J. *348 Pt 3*, 481–495.

Nystrom, M., and Mutanen, M. (2009). Diet and epigenetics in colon cancer. World J. Gastroenterol. *15*, 257–263.

Reddy, B.S. (2002). Types and amount of dietary fat and colon cancer risk: prevention by omega-3 fatty acid-rich diets. Environ. Health Prev. Med. *7*, 95–102.

Reya, T., Morrison, S.J., Clarke, M.F., and Weissman, I.L. (2001). Stem cells, cancer, and cancer stem cells. Nature *414*, 105–111.

Saigusa, S., Tanaka, K., Toiyama, Y., Yokoe, T., Okugawa, Y., Ioue, Y., Miki, C., and Kusunoki, M. (2009). Correlation of CD133, OCT4, and SOX2 in rectal cancer and their association with distant recurrence after chemoradiotherapy. Ann. Surg. Oncol. *16*, 3488–3498.

Sanchez, J.A., Krumroy, L., Plummer, S., Aung, P., Merkulova, A., Skacel, M., DeJulius, K.L., Manilich, E., Church, J.M., Casey, G., et al. (2009). Genetic and epigenetic classifications define clinical phenotypes and determine patient outcomes in colorectal cancer. Br. J. Surg. *96*, 1196–1204.

Schraivogel, D., Weinmann, L., Beier, D., Tabatabai, G., Eichner, A., Zhu, J.Y., Anton, M., Sixt, M., Weller, M., Beier, C.P., et al. (2011). CAMTA1 is a novel tumour suppressor regulated by miR-9/9* in glioblastoma stem cells. EMBO J. *30*, 4309–4322.

Szeto, W., Jiang, W., Tice, D.A., Rubinfeld, B., Hollingshead, P.G., Fong, S.E., Dugger, D.L., Pham, T., Yansura, D.G., Wong, T.A., et al. (2001). Overexpression of the retinoic acid-responsive gene Stra6 in human cancers and its synergistic induction by Wnt-1 and retinoic acid. Cancer Res. *61*, 4197–4205.

Vaiopoulos, A.G., Kostakis, I.D., Koutsilieris, M., and Papavassiliou, A.G. (2012). Colorectal cancer stem cells. Stem Cells *30*, 363–371.

van Engeland, M., Derks, S., Smits, K.M., Meijer, G.A., and Herman, J.G. (2011). Colorectal cancer epigenetics: complex simplicity. J. Clin. Oncol. *29*, 1382–1391.

Walther, A., Johnstone, E., Swanton, C., Midgley, R., Tomlinson, I., and Kerr, D. (2009). Genetic prognostic and predictive markers in colorectal cancer. Nat. Rev. Cancer *9*, 489–499.

Wicha, M.S., Liu, S., and Dontu, G. (2006). Cancer stem cells: an old idea—a paradigm shift. Cancer Res. *66*, 1883–1890, discussion 1895–1886.

Yang, Q., Graham, T.E., Mody, N., Preitner, F., Peroni, O.D., Zabolotny, J.M., Kotani, K., Quadro, L., and Kahn, B.B. (2005). Serum retinol binding protein 4 contributes to insulin resistance in obesity and type 2 diabetes. Nature *436*, 356–362.

Zeuner, A., Todaro, M., Stassi, G., and De Maria, R. (2014). Colorectal cancer stem cells: from the crypt to the clinic. Cell Stem Cell *15*, 692–705.

Stem Cell Reports, Volume *9*

Supplemental Information

RBP4-STRA6 Pathway Drives Cancer Stem Cell Maintenance and Mediates High-Fat Diet-Induced Colon Carcinogenesis

Sheelarani Karunanithi, Liraz Levi, Jennifer DeVecchio, George Karagkounis, Ofer Reizes, Justin D. Lathia, Matthew F. Kalady, and Noa Noy

Fig S1

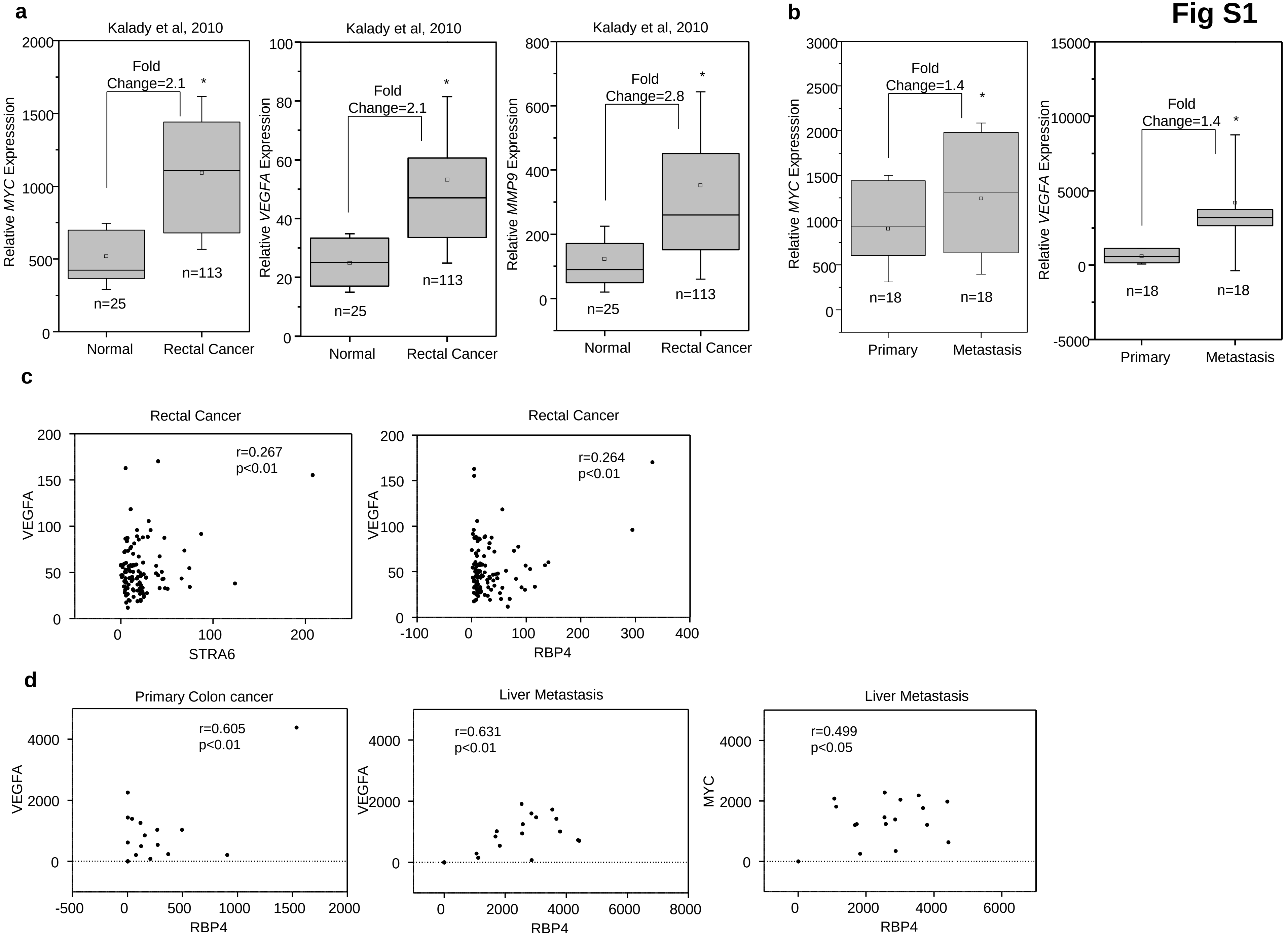
a
Kalady et al, 2010
Fold Change=2.1
*
Relative MYC Expresssion
n=25
n=113
Normal
Rectal Cancer
Kalady et al, 2010
Fold Change=2.1
Relative VEGFA Expression
n=25
n=113
Normal
Rectal Cancer
Kalady et al, 2010
Fold Change=2.8
Relative MMP9 Expression
n=25
n=113
Normal
Rectal Cancer
b
Fold Change=1.4
Relative MYC Expresssion
n=18
n=18
Primary
Metastasis
Fold Change=1.4
Relative VEGFA Expression
n=18
n=18
Primary
Metastasis
c
Rectal Cancer
r=0.267
p<0.01
VEGFA
STRA6
Rectal Cancer
r=0.264
p<0.01
VEGFA
RBP4
d
Primary Colon cancer
r=0.605
p<0.01
VEGFA
RBP4
Liver Metastasis
r=0.631
p<0.01
VEGFA
RBP4
Liver Metastasis
r=0.499
p<0.05
MYC
RBP4

Fig S2

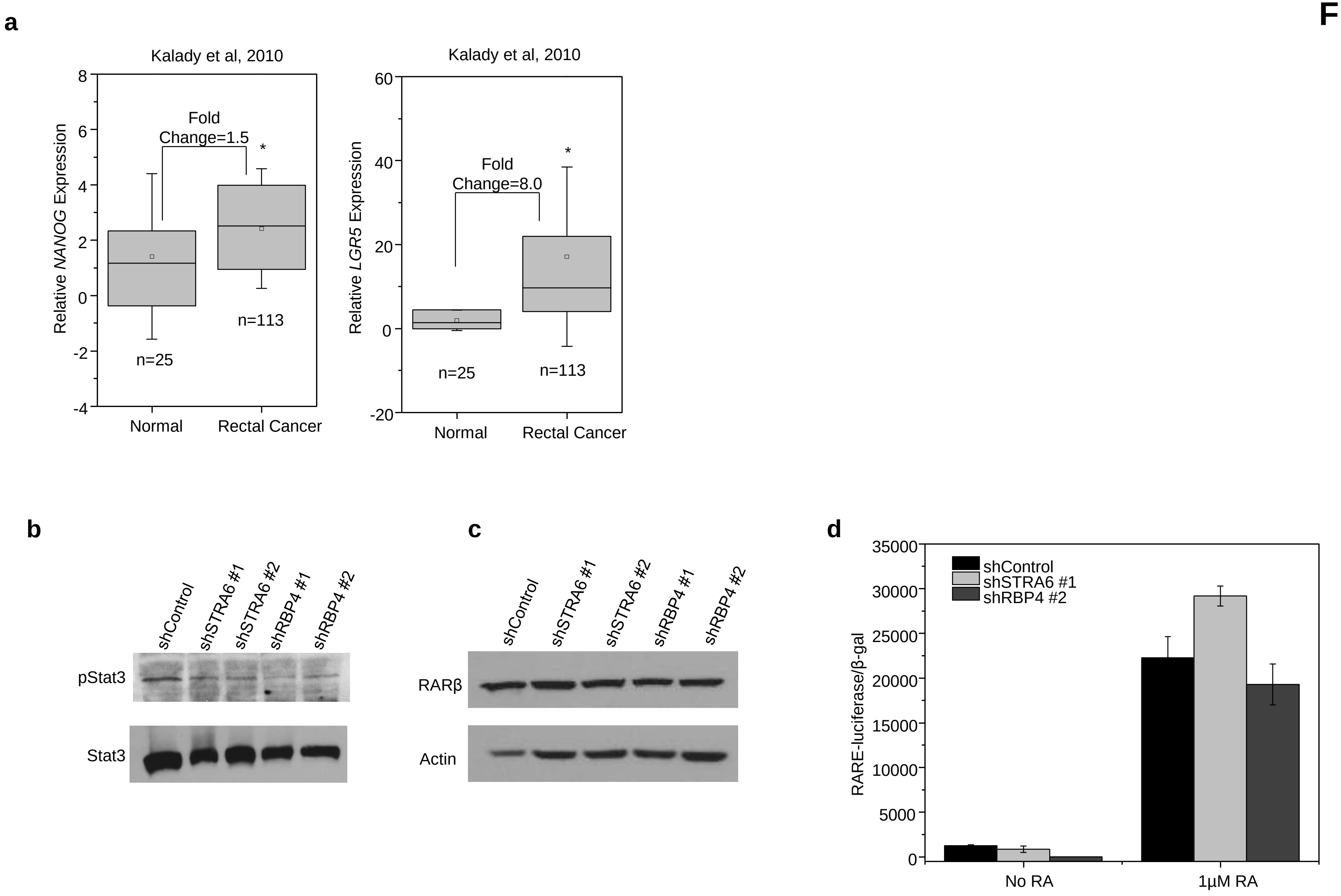
a
Kalady et al, 2010
Relative NANOG Expression
Fold Change=1.5
*
n=25
n=113
Normal
Rectal Cancer
Kalady et al, 2010
Relative LGR5 Expression
Fold Change=8.0
*
n=25
n=113
Normal
Rectal Cancer
b
shControl
shSTRA6 #1
shSTRA6 #2
shRBP4 #1
shRBP4 #2
pStat3
Stat3
c
shControl
shSTRA6 #1
shSTRA6 #2
shRBP4 #1
shRBP4 #2
RARβ
Actin
d
shControl
shSTRA6 #1
shRBP4 #2
RARE-luciferase/β-gal
No RA
1µM RA

Fig S3

a

SW480	Number of spheres positive wells/ Number of wells			
	Number of cells per well			
	1	2	5	10
shControl	8/24	13/24	21/24	24/24
shSTRA6 #1	3/24	4/24	12/24	16/24
shSTRA6 #2	3/24	5/24	14/24	19/24
shRBP4 #1	2/24	4/24	6/24	13/24
shRBP4 #2	4/24	7/24	9/24	12/24

b

SW480	Number of tumors / Number of injected mice				
	Number of cells per injection				
	$2.5*10^5$	$1.5*10^4$	$1*10^4$	5000	1500
shControl			4/5	2/5	1/5
shSTRA6 #1			2/5	0/5	0/5
shRBP4 #1	5/5	2/5			0/6

c

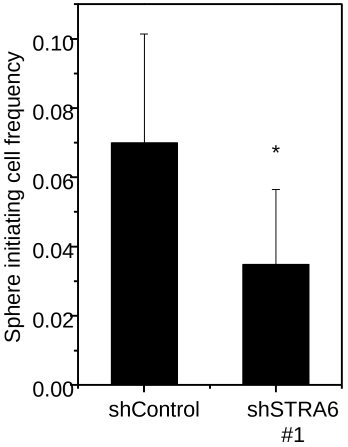

SW480	Number of spheres positive wells/ Number of wells					Sphere initiating-cell frequency		Fold Change	p value
	Number of cells per well					Estimate	95% Confidence Interval		
	1	2	5	10	20				
shControl	3/24	4/24	10/24	4/12	8/12	1/14.3	(1/9.86-1/20.7)		
shSTRA6 #1	1/24	2/24	2/24	5/12	6/12	1/28.7	(1/17.7-1/46.7)	2	$p<0.05$

d

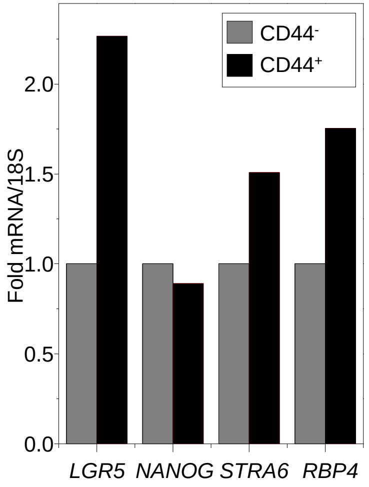

e

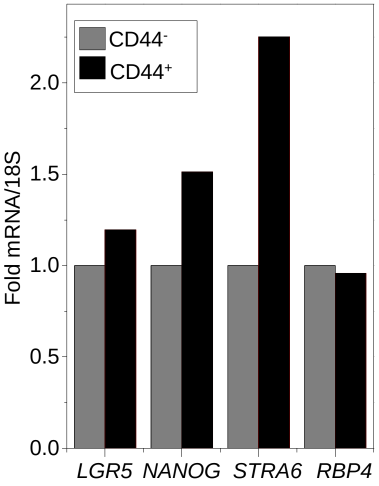

Fig S4

a

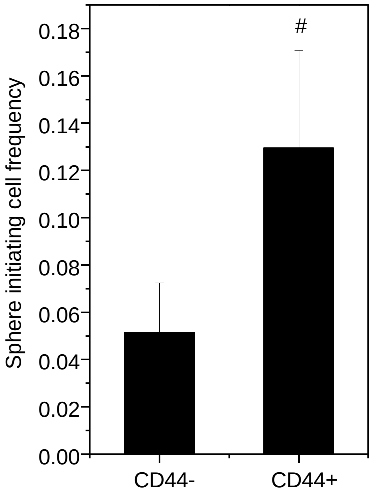

b

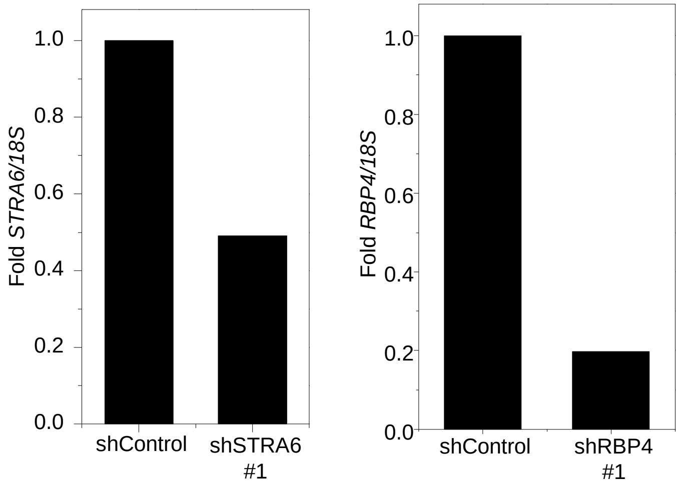

c

PDX656	Number of spheres positive wells/ Number of wells			
	Number of cells per well 1	5	10	20
shControl	7/24	13/24	22/24	24/24
shSTRA6 #1	3/24	10/24	14/24	20/24
shControl	7/24	14/24	19/24	21/24
shRBP4 #1	3/24	7/24	12/24	18/24

Fig S5

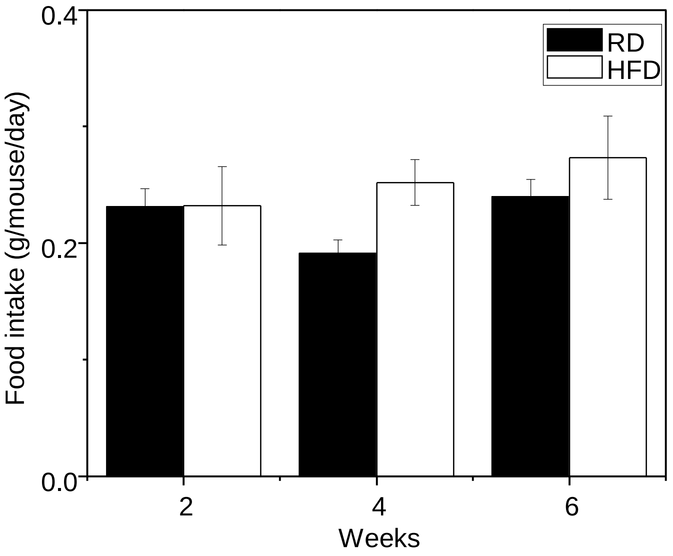

Supplemental Figure legends:

Figure S1: Related to Figure 1. JAK-STAT target gene expression correlate to RBP4-STRA6 pathway. a) Levels of STAT target genes, MYC, VEGFA and MMP9 in rectal cancer patients compared to normal tissue. * $p<0.01$ b) Analysis of expression levels of MYC and VEGFA in matched primary and metastatic colon cancer samples. *$p<0.05$ c) Correlation between expression levels of VEGFA and STRA6 or RBP4 in rectal cancer patients. Inset shows Pearson coefficient 'r' and p value. d) Correlation between expression levels of VEGFA and RBP4 in primary colon cancer and between VEGFA or MYC and RBP4 in matched liver metastasis patients are presented with Pearson coefficient 'r'.

Figure S2: Related to Figures 1 &2. RBP4-STRA6 pathway is related to colon cancer stemness. a) NANOG and LGR5 expression is upregulated in rectal cancer patients. *$p<0.01$. b) Immunoblots showing phospho-STAT3 and total STAT3 levels in SW480 cells stably knockdown for STRA6 or RBP4. c) Immunoblot showing unchanged protein levels of retinoic acid (RA) target gene, RARβ in SW480 cells stably expressing control, STRA6 or RBP4 shRNAs. Actin is used as a loading control. d) Activation of RARα in response to treatment with 1μM retinoic acid in SW480 stable lines was measured using transactivation assays. Cells were transfected with RAR-response element luciferase, RARα and β-gal constructs before treatments. Luciferase activity in response to treatment was normalized to β-galactosidase levels. Data are mean±SE of 3 biological replicates . $p<0.05$ (comparisons are between untreated and treated samples).

Figure S3: Related to Figure 4. Knockdown of STRA6 or RBP4 decrease CSC frequency. a) Limiting dilution assay in indicated SW480 lines shown in Fig 4B. Dose of cells seeded and the resulting frequency of sphere formation are tabulated. b) Tumor initiating cell frequency at indicated doses of SW480 cell injections from *in vivo* limiting dilution assay in Fig 4C. Frequency of tumor formation at respective dose of cells are shown in the table. c) *In vitro* limiting dilution assay of cells dissociated from SW480 tumors in Fig 4C expressing control or STRA6 shRNAs. Frequency estimates were calculated from 24 biological replicates of varying cell doses. Sphere re-initiation frequency calculated using ELDA software is plotted with error bars representing 95% confidence interval. *$p<0.05$. Stem cell frequency estimates within confidence intervals are shown in the table. d) Tumors from SW480 shControl injections were dissociated, stained with CD44-FITC antibody and sorted on BD FACS ARIA II. RNA levels of *STRA6*, *RBP4*, *NANOG* and *LGR5* in CD44 negative and positive populations are shown. Each bar represents expression analysis of cells dissociated after pooling several tumors from mice (n=5). e) Expression levels of *STRA6*, *RBP4*, *NANOG* and *LGR5* in CD44 negative and positive populations of PDX656 maintained as non-adherent spheres in culture.

Figure S4: Related to Figures 5. Knockdown efficiencies in a PDX. a) Sphere-initiating cell frequency of CD44 negative and positive cells sorted from cultured PDX 656 cells. Frequency estimates were calculated from 24 biological replicates of varying cell doses. Graph represents the fraction of sphere-forming stem cells and error bars represent 95% confidence intervals. #$p<0.01$. b) Knockdown efficiency of STRA6 (left) and RBP4 (right) shRNA in PDX 656 shown by differences in mRNA levels. c) Table shows dose of cells and frequency of sphere formation in a limiting dilution assay in PDX 656 performed in Fig 5A.

Figure S5: Related to Figures 6. Food intake is unaffected by diet. Food intake in mice fed a regular or high-fat diet in Fig 6A. Amount of food consumed normalized to body weights is shown during 2, 4 and 6 weeks after the mice were placed on the dietary regimen. n= 5 mice.
